# Regulation of PV interneuron plasticity by neuropeptide-encoding genes

**DOI:** 10.1038/s41586-025-08933-z

**Published:** 2025-04-30

**Authors:** Martijn Selten, Clémence Bernard, Diptendu Mukherjee, Fursham Hamid, Alicia Hanusz-Godoy, Fazal Oozeer, Christoph Zimmer, Oscar Marín

**Affiliations:** 1 Centre for Developmental Neurobiology, Institute of Psychiatry, Psychology and Neuroscience, King’s College London, London, UK; 2 Medical Research Council Centre for Neurodevelopmental Disorders, King’s College London, London, UK; 3 Present Address: Department of Clinical and Biomedical Sciences, Faculty of Health and Life Sciences, University of Exeter, Exeter, UK

**Keywords:** Cellular neuroscience, Synaptic plasticity

## Abstract

Neuronal activity must be regulated in a narrow permissive band for the proper operation of neural networks. Changes in synaptic connectivity and network activity—for example, during learning—might disturb this balance, eliciting compensatory mechanisms to maintain network function^1–3^. In the neocortex, excitatory pyramidal cells and inhibitory interneurons exhibit robust forms of stabilizing plasticity. However, although neuronal plasticity has been thoroughly studied in pyramidal cells^4–8^, little is known about how interneurons adapt to persistent changes in their activity. Here we describe a critical cellular process through which cortical parvalbumin-expressing (PV^+^) interneurons adapt to changes in their activity levels. We found that changes in the activity of individual PV^+^ interneurons drive bidirectional compensatory adjustments of the number and strength of inhibitory synapses received by these cells, specifically from other PV^+^ interneurons. High-throughput profiling of ribosome-associated mRNA revealed that increasing the activity of a PV^+^ interneuron leads to upregulation of two genes encoding multiple secreted neuropeptides: *Vgf* and *Scg2*. Functional experiments demonstrated that VGF is critically required for the activity-dependent scaling of inhibitory PV^+^ synapses onto PV^+^ interneurons. Our findings reveal an instructive role for neuropeptide-encoding genes in regulating synaptic connections among PV^+^ interneurons in the adult mouse neocortex.

## Abstract (web summary)

Increasing the activity of individual parvalbumin-expressing interneurons in the mouse brain modulates the number and strength of inhibitory parvalbumin synapses received by these cells through changes in the expression of the neuropeptide-encoding genes *Vgf* and *Scg2*.

## Main

Stabilizing network activity is essential for the operation of neural circuits, most critically during experience-dependent plasticity and learning^9,10^. Neurons regulate their activity through several mechanisms, including altering their intrinsic excitability and modulating the strength and number of synapses they receive^4–6,11,12^. The precise cellular and molecular mechanisms through which neurons undergo synaptic plasticity are, to a large extent, cell type-specific^13–15^.

In the cerebral cortex, the activity of individual neurons depends on the balance between synaptic excitation and inhibition, which is mediated by glutamatergic pyramidal cells and GABAergic (γ-aminobutyric acid-containing) interneurons, respectively. Pyramidal cells receive stable ratios of excitation and inhibition because any increase in excitation leads to a proportional increase in inhibition through the recruitment of inhibitory inputs^16,17^. This activity-dependent reorganization of inhibitory connections that target pyramidal cells requires changes in gene expression and is mediated by retrograde signals from the pyramidal cells^18–20^. Although interneurons are likely to exhibit similar forms of plasticity^21^, the extreme diversity of cortical GABAergic cells has limited their characterization. How individual interneurons adapt to changes in their activity remains largely unknown.

Fast-spiking basket cells that express the calcium-binding protein parvalbumin are the most abundant type of GABAergic interneuron in the mouse neocortex^22^. PV^+^ interneurons have a pivotal role in maintaining the excitation–inhibition balance of cortical circuits through potent perisomatic inhibition of pyramidal cells^23–26^. PV^+^ interneurons regulate experience-dependent plasticity^27^ and are involved in forming neuronal assemblies during learning^28,29^. Although it is evident that PV^+^ interneurons exhibit robust plasticity^30–33^, the cellular mechanisms and molecular signals regulating the adaptation of cortical PV^+^ interneurons to changes in their activity remain poorly characterized.

## Homeostatic scaling of inhibitory connectivity is bidirectional

**Fig. 1:**
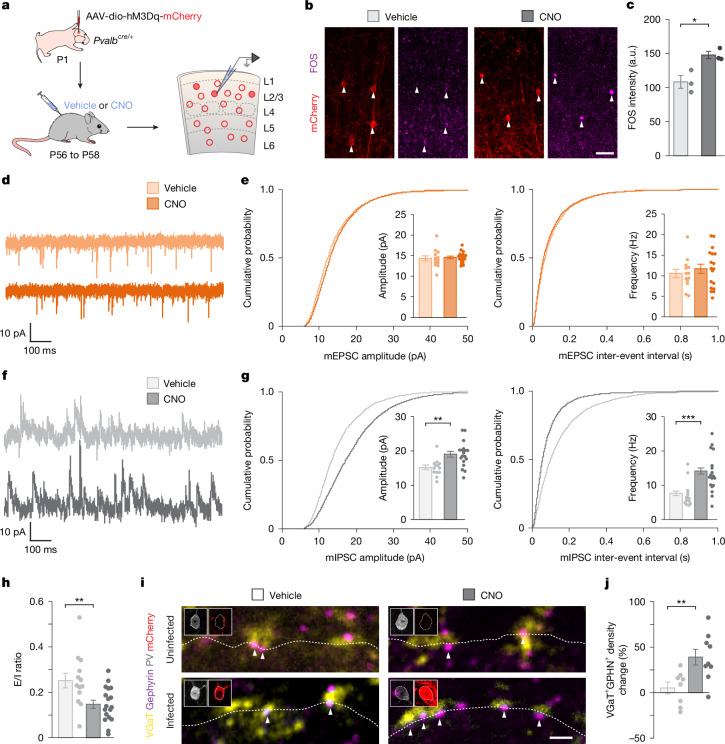
Inhibitory synapses mediate the homeostatic response of PV^+^ interneurons to increased activity. **a**, Experimental strategy. Adapted from ref. ^68^ (reprinted with permission from AAAS) and ref. ^69^. **b**, FOS and PV expression in coronal sections through S1. **c**, Quantification of FOS staining intensity in hM3Dq-mCherry-infected PV^+^ interneurons in vehicle- and CNO-treated mice (vehicle, *n* = 3 mice; CNO, *n* = 3 mice; two-tailed Student’s *t*-test, *P* = 0.02). a.u., arbitrary units. **d**, Traces of mEPSCs recorded from hM3Dq-mCherry-infected PV^+^ interneurons in vehicle- and CNO-treated mice. **e**, Amplitude (vehicle, *n* = 13 cells, 10 slices, 5 mice; CNO, *n* = 18 cells, 15 slices, 8 mice; two-tailed Student’s *t*-test, *P* = 0.71) and frequency (vehicle, *n* = 13 cells, 10 slices, 5 mice; CNO, *n* = 18 cells, 15 slices, 8 mice; two-tailed Student’s *t*-test, *P* = 0.48) of mEPSCs recorded from hM3Dq-mCherry-infected PV^+^ interneurons in vehicle- and CNO-treated mice. **f**, Traces of mIPSCs recorded from hM3Dq-mCherry-infected PV^+^ interneurons in vehicle- and CNO-treated mice. **g**, Amplitude (vehicle, *n* = 13 cells, 10 slices, 5 mice; CNO, *n* = 18 cells, 15 slices, 8 mice; two-tailed Student’s *t*-test, *P* = 0.003) and frequency (vehicle, *n* = 13 cells, 10 slices, 5 mice; CNO, *n* = 18 cells, 15 slices, 8 mice; Mann–Whitney *U* test, *P* = 0.001) of mIPSCs recorded from hM3Dq-mCherry-infected PV^+^ interneurons in vehicle- and CNO-treated mice. **h**, E/I ratio in hM3Dq-mCherry-infected PV^+^ interneurons in vehicle- and CNO-treated mice (vehicle, *n* = 13 cells, 10 slices, 5 mice; CNO, *n* = 18 cells, 15 slices, 8 mice; two-tailed Student’s *t*-test, *P* = 0.005). **i**, Presynaptic VGaT^+^ puncta and postsynaptic gephyrin^+^ clusters in infected and uninfected PV^+^ interneurons in vehicle- and CNO-treated mice. Insets show PV and mCherry immunoreactivity in cell somata. **j**, Quantification of the change in synaptic density between infected and uninfected PV^+^ interneurons in vehicle- and CNO-treated mice (vehicle, *n* = 8 mice; CNO, *n* = 9 mice; two-tailed Student’s *t*-test, *P* = 0.008). Data are mean ± s.e.m. Scale bar, 50 µm (**b**) and 1 µm (**i**). Source data

**Extended Data Fig. 1:**
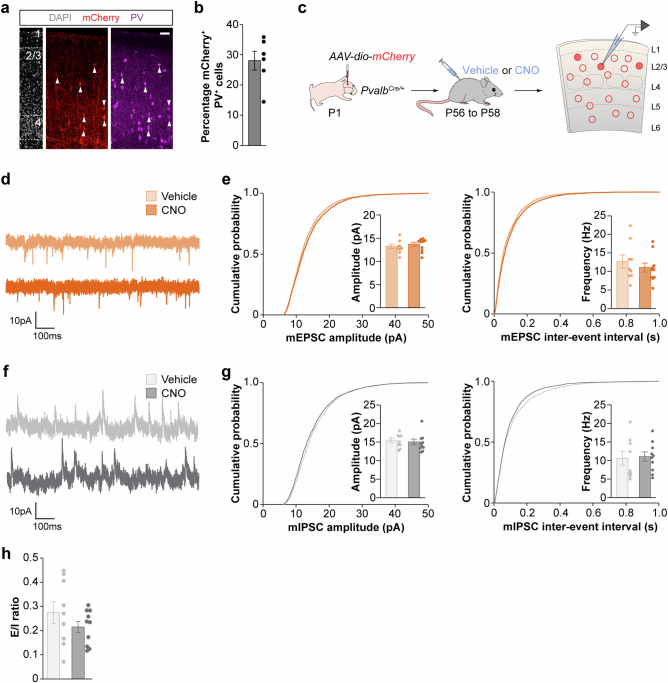
hM3Dq infection density and CNO control. **a**, mCherry and PV expression in coronal sections through S1. **b**, quantification of the percentage of mCherry^+^ PV^+^ interneurons in layer 2/3 (*n* = 6 mice). **c**, Experimental strategy. Adapted from ref. ^68^ (reprinted with permission from AAAS) and ref. ^69^. **d**, Traces of mEPSCs recorded from mCherry-infected PV+ interneurons in vehicle- and CNO-treated mice. **e**, Quantification of amplitude (vehicle, n = 9 cells, 9 slices, 3 mice; CNO, n = 10 cells, 6 slices, 3 mice; two-tailed Student’s t-test, p = 0.51) and frequency (vehicle, n = 9 cells, 9 slices, 3 mice; CNO, n = 10 cells, 6 slices, 3 mice; two-tailed Student’s t-test, p = 0.45) of mEPSCs recorded from mCherry-infected PV+ interneurons in vehicle- and CNO-treated mice. **f**, Traces of mIPSCs recorded from mCherry-infected PV+ interneurons in vehicle- and CNO-treated mice. **g**, Quantification of amplitude (vehicle, n = 9 cells, 9 slices, 3 mice; CNO, n = 10 cells, 6 slices, 3 mice; two-tailed Student’s t-test, p = 0.64) and frequency (vehicle, n = 9 cells, 9 slices, 3 mice; CNO, n = 10 cells, 6 slices, 3 mice; two-tailed Student’s t-test, p = 0.84) of mIPSCs recorded from mCherry-infected PV+ interneurons in vehicle- and CNO-treated mice. **h**, Excitation/Inhibition (E/I) ratio in mCherry-infected PV+ interneurons in vehicle- and CNO-treated mice (vehicle, n = 9 cells; CNO, n = 10 cells; two-tailed Student’s t-test, p = 0.24). Data are presented as mean ± s.e.m. Scale bar, 50 µm. Source data

Most research on stabilizing plasticity in the neocortex has relied on sensory deprivation paradigms, in which changes to network activity evoke changes in neuronal connectivity. Although these studies provided valuable insights into stabilization processes, manipulating network-wide activity prevents studying how individual neurons respond to changes in their activity. To circumvent this limitation, we directly activated a sparse population of PV^+^ interneurons. To this end, we used a chemogenetic approach based on designer receptors exclusively activated by designer drugs (DREADDs), which enables transient neuronal activation (hM3Dq) or inhibition (hM4Di) following administration of the pharmacologically inert molecule clozapine-*N*-oxide (CNO)^34^, targeting specifically neocortical PV^+^ interneurons. In brief, we injected layer 2/3 of the primary somatosensory cortex (S1) of neonatal *Pvalb*^*cre/+*^ mice with an adeno-associated virus (AAV) encoding Cre-dependent hM3Dq and mCherry to restrict its expression to PV^+^ interneurons (Fig. 1a). Most critically, we titrated the virus to infect only a relatively small fraction of PV^+^ interneurons in each mouse, thereby minimizing the possibility of indirect, network-induced changes (Extended Data Fig. 1a,b). We treated young adult mice with CNO or vehicle for two days and examined the synaptic inputs received by hM3Dq-expressing (hM3Dq^+^) PV^+^ interneurons using whole-cell electrophysiology. As a proxy of their activation, we measured FOS protein levels and found that hM3Dq^+^ PV^+^ cells contained higher levels of FOS in mice treated with CNO than in control mice (Fig. 1b,c). We also found that the amplitude and frequency of miniature excitatory postsynaptic currents (mEPSCs) recorded from hM3Dq^+^ PV^+^ interneurons were not affected by the transient activation of these cells (Fig. 1d,e). By contrast, both the amplitude and frequency of miniature inhibitory postsynaptic currents (mIPSCs) were increased following the activation of PV^+^ interneurons (Fig. 1f,g), which led to an overall reduction in the excitatory/inhibitory (E/I) ratio of these cells (Fig. 1h). This effect was caused by CNO-mediated activation of hM3Dq^+^ PV^+^ interneurons, as CNO treatment in mice injected with mCherry-expressing (mCherry^+^) AAVs did not affect the amplitude or frequency of mEPSCs and mIPSCs (Extended Data Fig. 1c–h).

**Extended Data Fig. 2:**
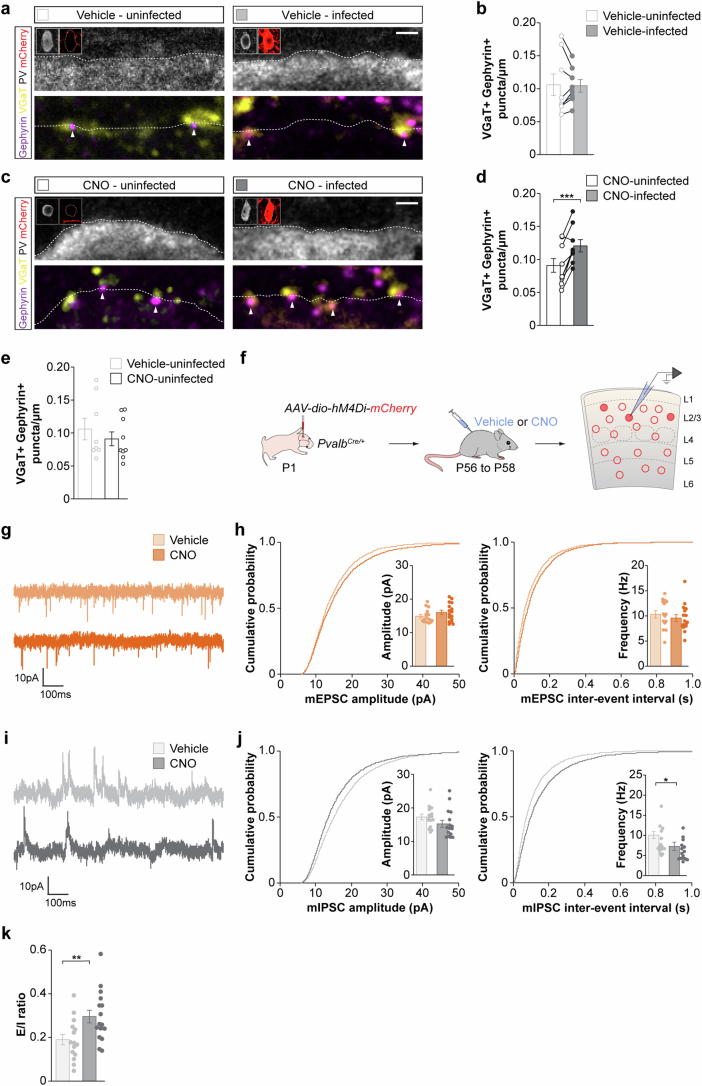
Bidirectional regulation of PV+ interneuron activity has opposite effects on inhibition. **a**, Presynaptic VGaT^+^ puncta and postsynaptic Gephyrin^+^ clusters in neighbouring uninfected and hM3Dq^+^ PV^+^ interneurons in vehicle-treated mice. Inserts show PV and mCherry immunoreactivity in cell somata. b, Quantification of the density of VGaT + /Gephyrin+ puncta on neighbouring uninfected and hM3D^+^ PV^+^ interneurons in vehicle-treated mice (*n* = 8; two-tailed paired Student’s t-test, *p* = 0.86). **c**, Presynaptic VGaT+ puncta and postsynaptic Gephyrin^+^ clusters in neighbouring uninfected and hM3Dq-mCherry+ PV+ interneurons in CNO-treated mice. Inserts show PV and mCherry immunoreactivity in the cell somata. **d**, Quantification of the density of VGaT + /Gephyrin+ puncta on neighbouring uninfected and hM3Dq-mCherry+ PV+ interneurons in CNO-treated mice (*n* = 9; two-tailed paired Student’s t-test, *p* = 8×10^−4^). **e**, Quantification of the density of VGaT^+^/Gephyrin^+^ puncta on neighbouring uninfected PV+ interneurons in vehicle-treated and CNO-treated mice (Vehicle: *n* = 8; CNO: n = 9; two-tailed Student’s t-test, *p* = 0.44). **f**, Experimental strategy. Adapted from ref. ^68^ (reprinted with permission from AAAS) and ref. ^69^. **g**, Traces of mEPSCs recorded from hM4Di-mCherry-infected PV+ interneurons in vehicle- and CNO-treated mice. **h**, Quantification of amplitude (vehicle, *n* = 15 cells, 14 slices, 7 mice; CNO, *n* = 16 cells, 12 slices, 7 mice; two-tailed Student’s t-test, *p* = 0.20) and frequency (vehicle, *n* = 15 cells, 14 slices, 7 mice; CNO, *n* = 16 cells, 12 slices, 7 mice; two-tailed Student’s t-test, *p* = 0.46) of mEPSCs recorded from hM3Dq-mCherry-infected PV+ interneurons in vehicle- and CNO-treated mice. **i**, Traces of mIPSCs recorded from hM4Di-mCherry-infected PV+ interneurons in vehicle- and CNO-treated mice. **j**, Quantification of amplitude (vehicle, *n* = 15 cells, 14 slices, 7 mice; CNO, *n* = 16 cells, 12 slices, 7 mice; two-tailed Student’s t-test, *p* = 0.14) and frequency (vehicle, *n* = 15 cells, 14 slices, 7 mice; CNO, *n* = 16 cells, 12 slices, 7 mice; two-tailed Student’s t-test, *p* = 0.03) of mIPSCs recorded from hM3Dq-mCherry-infected PV+ interneurons in vehicle- and CNO-treated mice. **k**, Excitation/Inhibition (E/I) ratio in hM4Di-mCherry infected PV interneurons in vehicle- and CNO-treated mice (vehicle, *n* = 15 cells, 14 slices, 7 mice; CNO, *n* = 16 cells, 12 slices, 7 mice; two-tailed Student’s t-test, *p* = 0.009). Data are presented as mean ± s.e.m. Scale bars, 1 µm. Source data

To investigate whether an increase in inhibitory synapses accompanied the increased inhibitory drive observed in activated PV^+^ interneurons, we performed immunohistochemistry labelling of presynaptic (VGaT) and postsynaptic (gephyrin) markers of inhibitory synapses and quantified their density on the soma of hM3Dq^+^ PV^+^ interneurons. The sparse infection allowed comparison of hM3Dq^+^ PV^+^ interneurons to neighbouring, uninfected PV^+^ interneurons as an internal control. We observed an increase in the density of inhibitory synaptic clusters contacting hM3Dq^+^ PV^+^ interneurons compared with uninfected neighbouring PV^+^ interneurons in CNO-treated but not vehicle-treated mice (Fig. 1i,j and Extended Data Fig. 2a–d). By contrast, CNO treatment did not affect uninfected PV^+^ interneurons (Extended Data Fig. 2e). These experiments revealed that the increase in inhibitory drive observed in hM3Dq^+^ PV^+^ interneurons is accompanied by an increase in the number of inhibitory synapses received by these cells. Moreover, this response is initiated by the increased activity in individual PV^+^ interneurons, as only activated, but not neighbouring control PV^+^ interneurons, showed increased synapse density.

Next, we investigated whether the inhibitory inputs received by PV^+^ interneurons change bidirectionally in response to changes in the activity of these cells. To this end, we reduced the activity of PV^+^ interneurons by expressing a Cre-dependent inhibitory DREADD (hM4Di; Extended Data Fig. 2f). Following CNO administration, we observed no changes in the amplitude and frequency of mEPSCs recorded from hM4Di^+^ PV^+^ interneurons (Extended Data Fig. 2g,h). By contrast, reducing the activity of PV^+^ interneurons led to a decrease in the frequency of mIPSCs recorded from these cells (Extended Data Fig. 2i,j), which resulted in an overall increase in the E/I ratio (Extended Data Fig. 2k). Together, these experiments revealed that modulating the activity of PV^+^ interneurons in vivo leads to a bidirectional compensatory change in the inhibitory drive these cells receive.

## Increased PV^+^ interneuron activity reorganizes synaptic connectivity

**Fig. 2:**
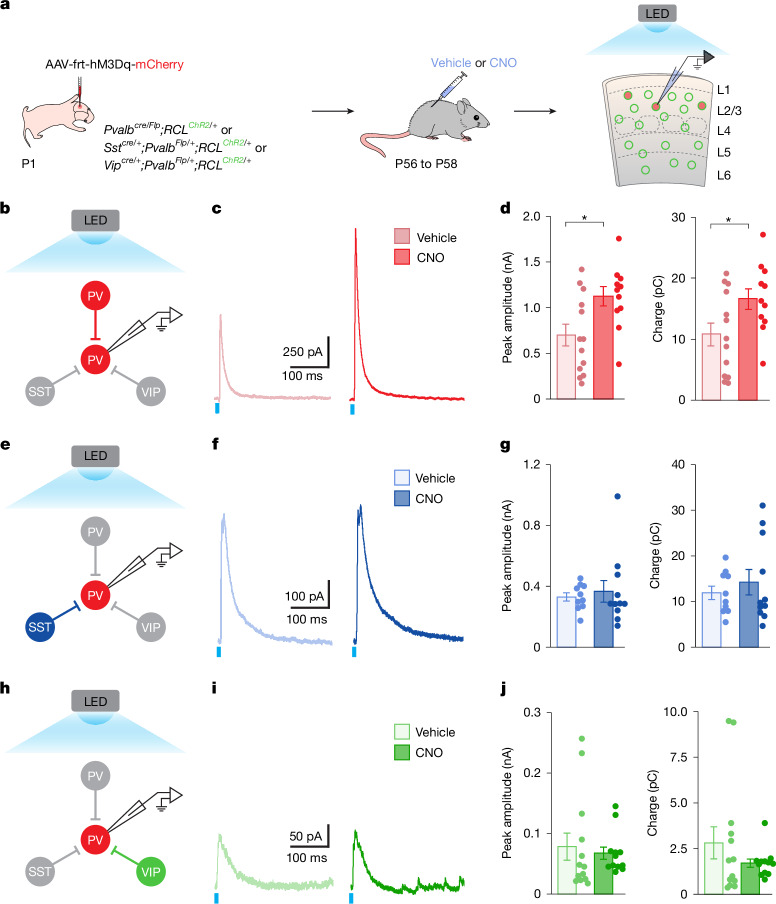
Homeostatic inhibition originates from PV^+^ interneurons. **a**, Experimental strategy. Adapted from ref. ^68^ (reprinted with permission from AAAS) and ref. ^69^. **b**, Recording and stimulation configuration in *Pvalb*^*cre/Flp*^*;RCL*^*Chr2/+*^ mice. **c**, Traces of evoked inhibitory postsynaptic currents (eIPSCs) recorded from hM3Dq^+^ PV^+^ interneurons following full-field stimulation of PV^+^ interneurons in vehicle- and CNO-treated mice. **d**, Peak amplitude (vehicle, *n* = 13 cells, 13 slices, 7 mice; CNO, *n* = 11 cells, 11 slices, 7 mice; two-tailed Student’s *t*-test, *P* = 0.02) and charge (vehicle, *n* = 13 cells, 13 slices, 7 mice; CNO, *n* = 11 cells, 11 slices, 7 mice; two-tailed Student’s *t*-test, *P* = 0.04) of LED-evoked eIPSCs. **e**, Recording and stimulation configuration in *Sst*^*cre/+*^*;Pvalb*^*Flp/+*^*;RCL*^*Chr2/+*^ mice. **f**, Traces of eIPSCs recorded from hM3Dq^+^ PV^+^ interneurons following full-field stimulation of SST^+^ interneurons in vehicle- and CNO-treated mice. **g**, Peak amplitude (vehicle, *n* = 10 cells, 10 slices, 6 mice; CNO, *n* = 11 cells, 11 slices, 5 mice; two-tailed Student’s *t*-test, *P* = 0.64) and charge (vehicle, *n* = 10 cells, 10 slices, 6 mice; CNO, *n* = 11 cells, 11 slices, 5 mice; two-tailed Student’s *t*-test, *P* = 0.48) LED-evoked eIPSCs. **h**, Recording and stimulation configuration in *Vip*^*cre/+*^*;Pvalb*^*Flp/+*^*;RCL*^*Chr2/+*^ mice. **i**, Traces of eIPSCs recorded from hM3Dq^+^ PV^+^ interneurons following full-field stimulation of VIP^+^ interneurons in vehicle- and CNO-treated mice. **j**, Peak amplitude (vehicle, *n* = 13 cells, 12 slices, 6 mice; CNO, *n* = 12 cells, 10 slices, 5 mice; two-tailed Student’s *t*-test, *P* = 0.67) and charge (vehicle, *n* = 13 cells, 12 slices, 6 mice; CNO, *n* = 12 cells, 10 slices, 5 mice; two-tailed Student’s *t*-test, *P* = 0.25) LED-evoked eIPSCs. Data are mean ± s.e.m. Source data

**Extended Data Fig. 3:**
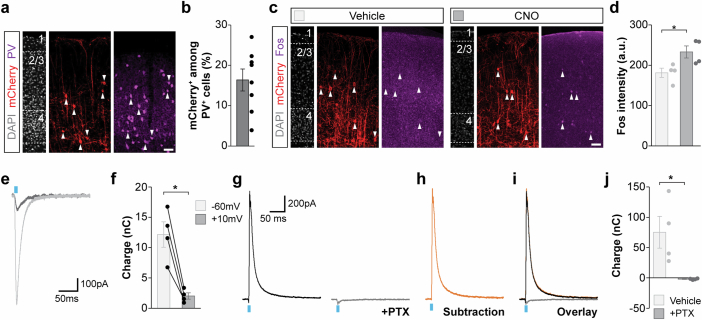
Validation of Flp-dependent hM3Dq-mCherry AAV and Channelrhodopsin control experiments. **a**, PV and mCherry expression in coronal sections through S1. **b**, Quantification of the percentage of mCherry+ PV+ interneurons in layer 2/3 (*n* = 8 mice). **c**, Fos and mCherry expression in coronal sections through S1 from vehicle- and CNO-treated mice. **d**, quantification of the intensity of Fos staining in hM3Dq+ PV+ interneurons in vehicle- and CNO-treated mice (vehicle, *n* = 4 mice; CNO, *n* = 4 mice; two-tailed Student’s t-test, *p* = 0.03). **e**, Traces of Channelrhodopsin (ChR2) evoked currents recorded from hM3Dq-mCherry expressing PV+ interneurons at a holding voltage of −60 mV and +10 mV in the presence of picrotoxin (PTX) at 100% LED power. **f**, Quantification of ChR2-evoked charge in the presence of PTX (−60 mV, n = 4 cells; +10 mV, n = 4 cells; two-tailed paired Student’s t-test, p = 0.01). **g**, Traces of eIPSCs recorded from an hM3Dq+ PV+ interneuron at a holding voltage of +10 mV following stimulation of ChR2 expressing PV+ interneurons at 100% LED power in the absence or presence of picrotoxin (PTX). The PTX condition reflects the contribution of direct ChR2-evoked current (compare to panel a). **h**, The resultant trace of the subtraction of the traces is shown in panel (g). **i**, Overlay of panels (g) and (h). **j**, Quantification of eIPSC charges recorded from hM3Dq+ PV+ interneurons following stimulation of ChR2+ PV+ interneurons at 100% LED power in the absence or presence of PTX (control, n = 4 cells; PTX, n = 4 cells; two-tailed Student’s t-test, p = 0.03). Blue boxes indicate LED stimulation. Data are presented as mean ± s.e.m. Scale bars, 50 µm. Source data

Changes in the number of inhibitory inputs dynamically follow the activity of PV^+^ cells. We next tested whether these inputs originate from a specific interneuron population. PV^+^ interneurons receive synapses from the three largest subclasses of interneurons: other PV^+^ interneurons, somatostatin-expressing (SST^+^) interneurons and vasoactive intestinal peptide-expressing (VIP^+^) interneurons^35,36^. To determine the contribution of each of these populations to the changes in presynaptic inhibitory input that follow the increased activity of PV^+^ interneurons, we used optogenetic stimulation to evoke synaptic output from each subclass of interneuron (PV^+^, SST^+^ or VIP^+^) while recording from hM3Dq^+^ PV^+^ interneurons (Fig. 2a). To this end, we first generated mice expressing flippase (Flp) recombinase under the *Pvalb* locus along with Cre-dependent channelrhodopsin (ChR2) alleles (*Pvalb*^*Flp/Flp*^*;RCL*^*ChR2/Chr2*^), and then crossed these mice with *Pvalb*^*cre/+*^, *Sst*^*cre/+*^ and *Vip*^*cre/+*^ mice to generate mice in which ChR2 was expressed in one of each of the three subclasses of interneurons (*Pvalb*^*cre/*Flp^*;RCL*^*ChR2/+*^, *Sst*^*cre/+*^*;Pvalb*^*Flp/+*^*;RCL*^*ChR2/+*^ and *Vip*^*cre/+*^*;Pvalb*^*Flp/+*^*;RCL*^*ChR2/+*^). To modify the activity of PV^+^ interneurons in these experiments, we injected these mice with AAVs encoding a Flp-dependent hM3Dq (Fig. 2a). As in previous experiments, we titrated the virus to infect a small fraction of PV^+^ interneurons (Extended Data Fig. 3a,b) and verified its effectiveness to activate hM3Dq^+^ PV^+^ interneurons using FOS (Extended Data Fig. 3c,d).

**Extended Data Fig. 4:**
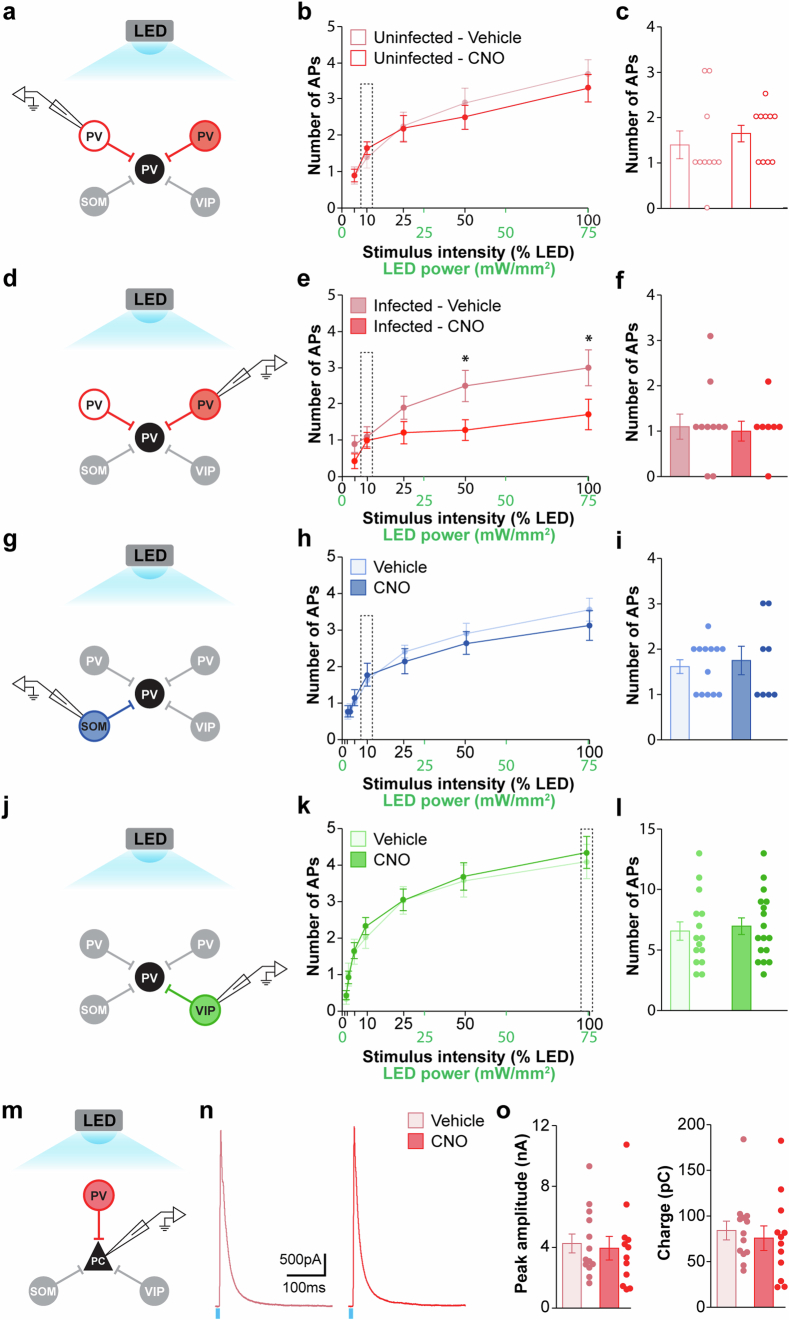
Excitability of channelrhodopsin-expressing interneurons following LED stimulation at different intensities. **a**, Experimental strategy to record the excitability of uninfected PV^+^ interneurons in a cell-attached configuration in vehicle- and CNO-treated *Pvalb*^*Cre/Flp*^*;RCL*^*Chr2/+*^ mice. **b**, Quantification of the number of elicited action potentials (APs) following LED stimulation at different intensities in uninfected PV^+^ interneurons from vehicle- and CNO-treated mice (5% LED-intensity, vehicle, *n* = 10 cells, 9 slices, 5 mice; CNO, *n* = 11 cells, 10 slices, 7 mice; two-tailed Student’s t-test, *p* = 0.97; 10% LED-intensity, vehicle, *n* = 10 cells, 9 slices, 5 mice; CNO, *n* = 11 cells, 10 slices, 7 mice; two-tailed Student’s t-test, *p* = 0.59; 25% LED-intensity, vehicle, *n* = 10 cells, 9 slices, 5 mice; CNO, *n* = 11 cells, 10 slices, 7 mice; two-tailed Student’s t-test, *p* = 0.76; 50% LED-intensity, vehicle, *n* = 10 cells, 9 slices, 5 mice; CNO, *n* = 11 cells, 10 slices, 7 mice; two-tailed Student’s t-test, *p* = 0.32; 100% LED-intensity, vehicle, *n* = 10 cells, 9 slices, 5 mice; CNO, *n* = 11 cells, 10 slices, 7 mice; two-tailed Student’s t-test, *p* = 0.29). **c**, Quantification of the selected stimulation intensity from panel (b). **d**, Experimental strategy to record the excitability of infected PV+ interneurons in a cell-attached configuration in vehicle- and CNO-treated *Pvalb*^*Cre/Flp*^*;RCL*^*Chr2/+*^ mice. **e**, Quantification of the number of elicited APs following LED stimulation at different intensities in infected PV^+^ interneurons from vehicle- and CNO-treated mice (5% LED-intensity, vehicle, *n* = 10 cells, 9 slices, 5 mice; CNO, *n* = 11 cells, 10 slices, 7 mice; two-tailed Student’s t-test, *p* = 0.23; 10% LED-intensity, vehicle, *n* = 10 cells, 9 slices, 5 mice; CNO, *n* = 11 cells, 10 slices, 7 mice; two-tailed Student’s t-test, *p* = 1.00; 25% LED-intensity, vehicle, *n* = 10 cells, 9 slices, 5 mice; CNO, *n* = 11 cells, 10 slices, 7 mice; two-tailed Student’s t-test, *p* = 0.21; 50% LED-intensity, vehicle, *n* = 10 cells, 9 slices, 5 mice; CNO, *n* = 11 cells, 10 slices, 7 mice; two-tailed Student’s t-test, *p* = 0.04; 100% LED-intensity, vehicle, *n* = 10 cells, 9 slices, 5 mice; CNO, *n* = 11 cells, 10 slices, 7 mice; two-tailed Student’s t-test, *p* = 0.04). **f**, Quantification of the selected stimulation intensity from panel (e). **g**, Experimental strategy to record the excitability of SST^+^ interneurons in a cell-attached configuration in vehicle- and CNO-treated *Sst*^*Cre/+*^*;Pvalb*^*Flp/+*^*;RCL*^*Chr2/+*^ mice. **h**, Quantification of the number of elicited APs following LED stimulation at different intensities in SOM^+^ interneurons in vehicle- and CNO-treated mice (2% LED-intensity, vehicle, *n* = 13 cells, 8 slices, 5 mice; CNO, *n* = 8 cells, 5 slices, 2 mice; two-tailed Student’s t-test, *p* = 0.65; 3% LED-intensity, vehicle, *n* = 13 cells, 8 slices, 5 mice; CNO, *n* = 8 cells, 5 slices, 2 mice; two-tailed Student’s t-test, *p* = 0.61; 5% LED-intensity, vehicle, *n* = 13 cells, 8 slices, 5 mice; CNO, *n* = 8 cells, 5 slices, 2 mice; two-tailed Student’s t-test, *p* = 0.59; 10% LED-intensity, vehicle, *n* = 13 cells, 8 slices, 5 mice; CNO, *n* = 8 cells, 5 slices, 2 mice; two-tailed Student’s t-test, *p* = 0.67; 25% LED-intensity, vehicle, *n* = 13 cells, 8 slices, 5 mice; CNO, *n* = 8 cells, 5 slices, 2 mice; two-tailed Student’s t-test, *p* = 0.48; 50% LED-intensity, vehicle, *n* = 13 cells, 8 slices, 5 mice; CNO, *n* = 8 cells, 5 slices, 2 mice; two-tailed Student’s t-test, *p* = 0.55; 100% LED-intensity, vehicle, *n* = 13 cells, 8 slices, 5 mice; CNO, *n* = 8 cells, 5 slices, 2 mice; two-tailed Student’s t-test, *p* = 0.42). **i**, Quantification of the selected stimulation intensity from panel (h). **j**, Experimental strategy to record the excitability of VIP^+^ interneurons in a cell-attached configuration in vehicle- and CNO-treated *Vip*^*Cre/+*^*;Pvalb*^*Flp/+*^*;RCL*^*Chr2/+*^ mice. **k**, Quantification of the number of elicited APs following LED stimulation at different intensities in VIP^+^ interneurons in vehicle- and CNO-treated mice (2% LED-intensity, vehicle, *n* = 15 cells, 10 slices, 3 mice; CNO, *n* = 17 cells, 10 slices, 3 mice; two-tailed Student’s t-test, *p* = 0.56; 3% LED-intensity, vehicle, *n* = 15 cells, 10 slices, 3 mice; CNO, *n* = 17 cells, 10 slices, 3 mice; two-tailed Student’s t-test, *p* = 0.77; 5% LED-intensity, vehicle, *n* = 15 cells, 10 slices, 3 mice; CNO, *n* = 17 cells, 10 slices, 3 mice; two-tailed Student’s t-test, *p* = 0.94; 10% LED-intensity, vehicle, *n* = 15 cells, 10 slices, 3 mice; CNO, *n* = 17 cells, 10 slices, 3 mice; two-tailed Student’s t-test, *p* = 0.38; 25% LED-intensity, vehicle, *n* = 15 cells, 10 slices, 3 mice; CNO, *n* = 17 cells, 10 slices, 3 mice; two-tailed Student’s t-test, *p* = 0.98; 50% LED-intensity, vehicle, *n* = 15 cells, 10 slices, 3 mice; CNO, *n* = 17 cells, 10 slices, 3 mice; two-tailed Student’s t-test, *p* = 0.85; 100% LED-intensity, vehicle, *n* = 15 cells, 10 slices, 3 mice; CNO, *n* = 17 cells, 10 slices, 3 mice; two-tailed Student’s t-test, *p* = 0.69). **l**, Quantification of the selected stimulation intensity from panel (k). **m**, Experimental strategy to record the inhibitory inputs from infected PV+ interneurons in vehicle- and CNO-treated *Pvalb*^*Cre/Flp*^*;RCL*^*Chr2/+*^ mice from pyramidal neurons. n, Traces of eIPSCs recording from layer 2/3 pyramidal cells following full-field stimulation of PV+ interneurons in vehicle- and CNO-treated *Pvalb*^*Cre/Flp*^*;RCL*^*Chr2/+*^ mice. **o**, Quantification of the peak amplitude (vehicle, *n* = 13 cells, 13 slices, 5 mice; CNO, *n* = 12 cells, 12 slices, 5 mice; two-tailed Student’s t-test, *p* = 0.76) and charge (vehicle, *n* = 13 cells, 13 slices, 5 mice; CNO, *n* = 12 cells, 12 slices, 5 mice; two-tailed Student’s t-test, *p* = 0.66) of eIPSCs evoked by LED stimulation in layer 2/3 pyramidal cells following full-field stimulation of PV^+^ interneurons in vehicle- and CNO-treated *Pvalb*^*Cre/Flp*^*;RCL*^*Chr2/+*^ mice. Data are presented as mean ± s.e.m. Dotted boxes indicate the selected stimulation strength. Source data

**Extended Data Fig. 5:**
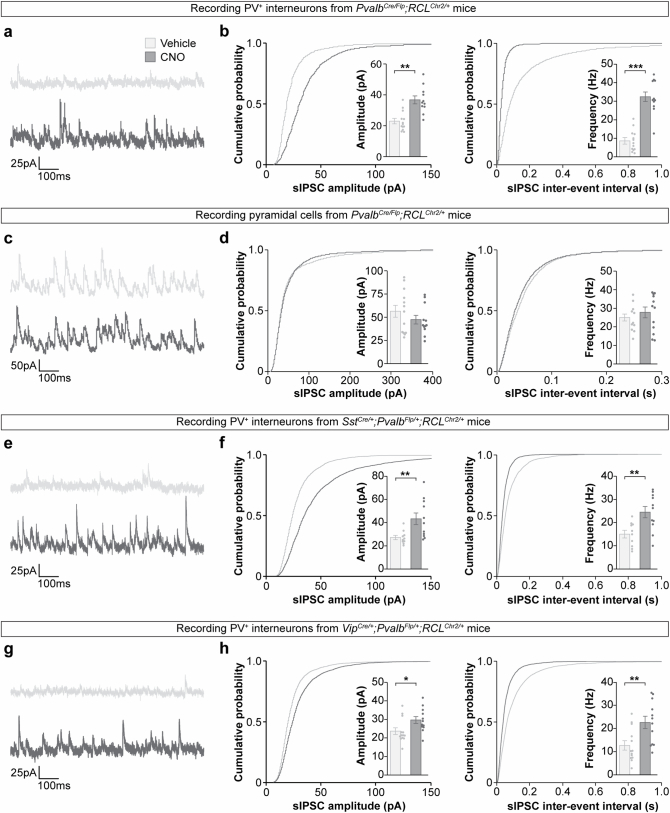
Spontaneous IPSC recordings confirm the increase in PV^+^ interneuron activity. **a**, Traces of spontaneous IPSCs (sIPSCs) recorded from hM3Dq^+^ PV^+^ interneurons in vehicle- and CNO-treated *Pvalb*^*Cre/Flp*^*;RCL*^*Chr2/+*^ mice. **b**, Quantification of amplitude (vehicle, *n* = 13 cells, 13 slices, 7 mice; CNO, *n* = 11 cells, 11 slices, 7 mice; two-tailed Student’s t-test, *p* = 0.003) and frequency vehicle, *n* = 13 cells, 13 slices, 7 mice; CNO, *n* = 11 cells, 11 slices, 7 mice; two-tailed Student’s t-test, *p* = 1.1×10^−7^) of sIPSCs recorded from hM3Dq^+^ PV^+^ interneurons in vehicle- and CNO-treated *Pvalb*^*Cre/Flp*^*;RCL*^*Chr2/+*^ mice. **c**, Traces of sIPSCs recorded from layer 2/3 pyramidal neurons in vehicle- and CNO-treated *Pvalb*^*Cre/Flp*^*;RCL*^*Chr2/+*^ mice. **d**, Quantification of amplitude (vehicle, *n* = 13 cells, 13 slices, 5 mice; CNO, *n* = 12 cells, 12 slices, 5 mice; two-tailed Student’s t-test, *p* = 0.28) and frequency (vehicle, *n* = 13 cells, 13 slices, 5 mice; CNO, *n* = 12 cells, 12 slices, 5 mice; two-tailed Student’s t-test, *p* = 0.43) of sIPSCs recorded from layer 2/3 pyramidal neurons in vehicle- and CNO-treated *Pvalb*^*Cre/Flp*^*;RCL*^*Chr2/+*^ mice. **e**, Traces of sIPSCs recorded from hM3Dq^+^ PV^+^ interneurons in vehicle- and CNO-treated *Sst*^*Cre/+*^*;Pvalb*^*Flp/+*^*;RCL*^*Chr2/+*^ mice. **f**, Quantification of amplitude (vehicle, *n* = 10 cells, 10 slices, 5 mice; CNO, *n* = 11 cells, 11 slices, 5 mice; two-tailed Student’s t-test, *p* = 0.009) and frequency (vehicle, *n* = 10 cells, 10 slices, 5 mice; CNO, *n* = 11 cells, 11 slices, 5 mice; two-tailed Student’s t-test, *p* = 0.005) of sIPSCs recorded from hM3Dq^+^ PV^+^ interneurons in vehicle- and CNO-treated *Sst*^*Cre/+*^*;Pvalb*^*Flp/+*^*;RCL*^*Chr2/+*^ mice. **g**, Traces of sIPSCs recorded from hM3Dq^+^ PV^+^ interneurons in vehicle- and CNO-treated *Vip*^*Cre/+*^*;Pvalb*^*Flp/+*^*;RCL*^*Chr2/+*^ mice. **h**, Quantification of amplitude (vehicle, *n* = 13 cells, 12 slices, 6 mice; CNO, *n* = 12 cells, 10 slices, 5 mice; two-tailed Student’s t-test, *p* = 0.03) and frequency (vehicle, *n* = 13 cells, 12 slices, 6 mice; CNO, *n* = 12 cells, 10 slices, 5 mice; two-tailed Student’s t-test, *p* = 0.006) of sIPSCs recorded from hM3Dq^+^ PV^+^ interneurons in vehicle- and CNO-treated *Vip*^*Cre/+*^*;Pvalb*^*Flp/+*^*;RCL*^*Chr2/+*^ mice. Data are presented as mean ± s.e.m. Source data

**Extended Data Fig. 6:**
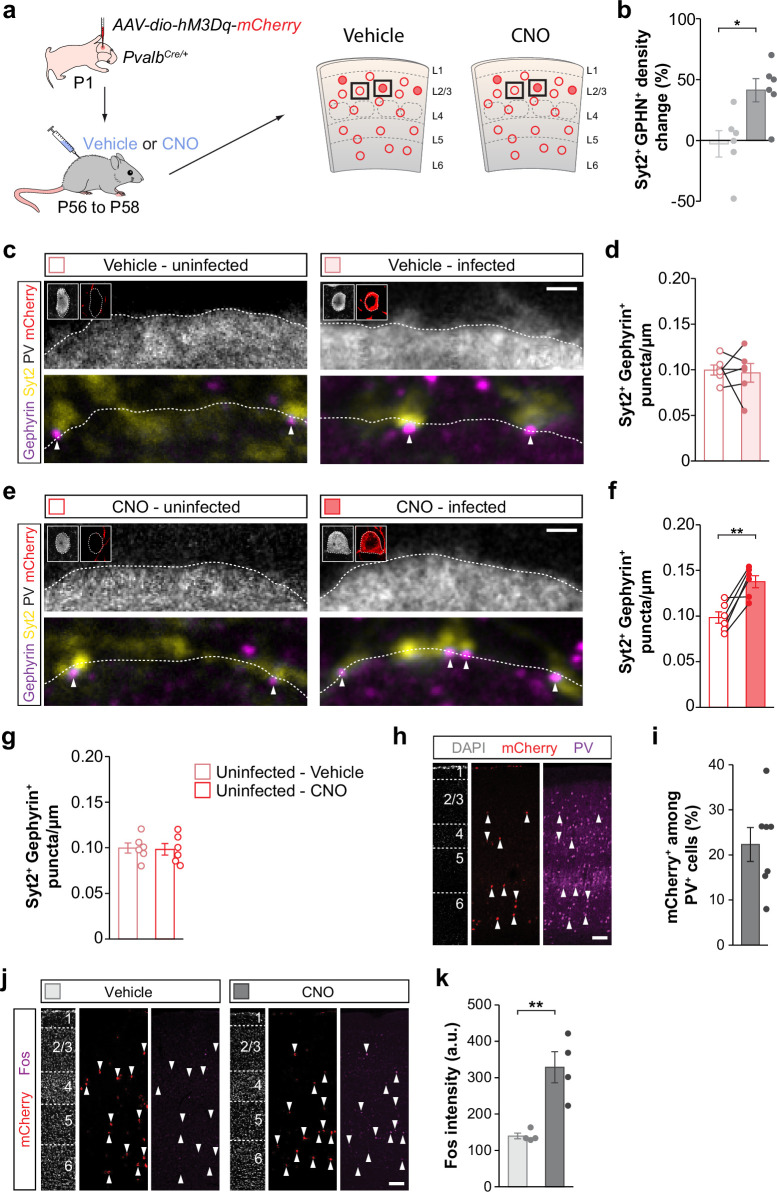
Increasing the activity of PV^+^ interneurons leads to an increase in inhibitory synaptic density. **a**, Schematic outline of the experimental strategy. Adapted from ref. ^68^ (reprinted with permission from AAAS) and ref. ^69^. **b**, Quantification of change in synaptic density between infected and uninfected PV^+^ interneurons in vehicle- and CNO-treated mice (vehicle, *n* = 6 mice; CNO, *n* = 6 mice; two-tailed Student’s t-test, *p* = 0.01). **c**, Confocal images illustrating presynaptic Syt2^+^ puncta and postsynaptic Gephyrin^+^ clusters in neighbouring uninfected and hM3Dq-mCherry expressing PV^+^ interneurons in vehicle-treated mice. Inserts show PV and mCherry immunoreactivity of the cell’s soma. **d**, Quantification of the density of colocalising Syt2^+^/Gephyrin^+^ puncta on neighbouring uninfected and hM3Dq expressing PV^+^ interneurons in vehicle-treated mice (*n* = 6; two-tailed paired Student’s t-test, *p* = 0.79). **e**, Confocal images illustrating presynaptic Syt2^+^ puncta and postsynaptic Gephyrin^+^ clusters in neighbouring uninfected and hM3Dq-mCherry expressing PV^+^ interneurons in CNO-treated mice. Inserts show PV and mCherry immunoreactivity of the cell’s soma. **f**, Quantification of the density of colocalising Syt2^+^/Gephyrin^+^ puncta on neighbouring uninfected and hM3Dq expressing PV^+^ interneurons in CNO-treated mice (*n* = 6; two-tailed paired Student’s t-test, *p* = 0.006). **g**, Quantification of the density of Syt2^+^/Gephyrin^+^ puncta on neighbouring uninfected PV^+^ interneurons in vehicle-treated and CNO-treated mice (vehicle: *n* = 6; CNO: *n* = 6; two-tailed Student’s t-test, *p* = 0.87). **h**, Validation of *HA-vTRAP-hM3Dq-myc* AAV showing PV and mCherry expression in coronal sections through S1. **i**, Quantification of the percentage of mCherry^+^ PV^+^ interneurons in layer 2/3 (*n* = 7 mice). **j**, Fos and mCherry expression in coronal sections through S1 from vehicle- and CNO-treated mice. **k**, Quantification of the intensity of Fos staining in HA-vTRAP-hM3Dq-myc infected PV^+^ interneurons in vehicle- and CNO-treated mice (vehicle, *n* = 4 mice; CNO, *n* = 4 mice; two-tailed Student’s t-test, *p* = 0.004). Scale bar, 1 µm (c and e) and 50 µm (h and j). Data are presented as mean ± s.e.m. Source data

We first examined the contribution of other PV^+^ interneurons to the increased inhibitory drive observed in hM3Dq^+^ PV^+^ interneurons following their increased activity (Fig. 2b). Because ChR2 is also expressed in the recorded cells from *Pvalb*^*cre/Flp*^*;RCL*^*ChR2/+*^ mice, we performed control whole-cell patch-clamp recordings from synaptically isolated PV^+^ interneurons to confirm that the contribution of ChR2-mediated currents is negligible compared with the evoked synaptic currents in our recording conditions (Extended Data Fig. 3e–j). We tested whether CNO treatment affected the excitability of both infected and uninfected PV^+^ interneurons in response to ChR2 stimulation (Extended Data Fig. 4a–f). Whereas infected PV^+^ interneurons in CNO-treated mice exhibited reduced excitability at higher LED stimulus intensities, stimulation at 10% LED power (7.5 mW mm^−2^) led to the same number of evoked action potentials as in infected PV^+^ interneurons from vehicle-treated mice (Extended Data Fig. 4e,f). We recorded spontaneous inhibitory postsynaptic currents (sIPSCs) from each cell before the ChR2 stimulation to verify the increase in inhibitory inputs following treatment with CNO. As expected from previous experiments, we observed increased amplitude and frequency of sIPSCs in hM3Dq^+^ PV^+^ interneurons from CNO-treated compared to vehicle-treated *Pvalb*^*cre/Flp*^*;RCL*^*ChR2/+*^ mice (Extended Data Fig. 5a,b). ChR2-mediated stimulation of PV^+^ interneurons also revealed an increase in the peak amplitude and charge of ChR2-evoked inhibitory postsynaptic currents recorded from hM3Dq^+^ PV^+^ interneurons in CNO-treated mice compared with controls (Fig. 2c,d). Of note, this increase was not due to the global presynaptic strengthening of all PV^+^ synapses because we detected no changes in sIPSCs or ChR2-evoked synaptic currents when recording from pyramidal cells (Extended Data Figs. 4m–o and  5c,d). These experiments demonstrated that the increased inhibition received by PV^+^ interneurons following their prolonged activation derives, at least in part, from other PV^+^ interneurons. To corroborate this observation, we quantified the number of synapses made by PV^+^ interneurons on hM3Dq^+^ PV^+^ interneurons and uninfected neighbouring cells using the PV^+^ specific presynaptic marker synaptotagmin-2 (SYT2)^37^. We found an increase in the density of PV^+^ synaptic clusters contacting hM3Dq^+^ PV^+^ interneurons compared with uninfected neighbouring PV^+^ interneurons in CNO-treated but not vehicle-treated mice (Extended Data Fig. 6a–f). Notably, the density of PV^+^ synaptic clusters was indistinguishable between uninfected cells from vehicle- and CNO-treated mice (Extended Data Fig. 6g). Together, these experiments revealed that increasing the activity of individual PV^+^ interneurons leads to a homeostatic increase in the number of inhibitory synapses these cells receive from other PV^+^ interneurons.

We next tested whether SST^+^ and VIP^+^ interneurons also contribute to the increased inhibitory drive observed in hM3Dq^+^ PV^+^ interneurons following increased activity using the same strategy (Fig. 2a). First, we verified that CNO treatment did not affect the excitability of SOM^+^ or VIP^+^ interneurons (Extended Data Fig. 4g–l). sIPSCs recorded from the hM3Dq^+^ PV^+^ interneurons revealed an increase in amplitude and frequency in CNO-treated mice from both *Sst*^*cre/+*^*;Pvalb*^*Flp/+*^*;RCL*^*ChR2/+*^ and *Vip*^*cre/+*^*;Pvalb*^*Flp/+*^*;RCL*^*ChR2/+*^ mice, which confirmed that CNO treatment in these experiments also led to an increase in inhibitory inputs contacting hM3Dq^+^ PV^+^ interneurons (Extended Data Fig. 5e–h). However, we observed no changes between CNO-treated mice and controls when recording ChR2-evoked synaptic currents in the same PV^+^ interneurons following optogenetic stimulation of SST^+^ or VIP^+^ interneurons (Fig. 2e–j). Together, these experiments demonstrated that raising the activity of PV^+^ interneurons increases the strength and number of inhibitory inputs these cells receive from other PV^+^ interneurons (PV–PV connectivity) but not those from SST^+^ or VIP^+^ interneurons.

## Activity-dependent gene expression

**Fig. 3:**
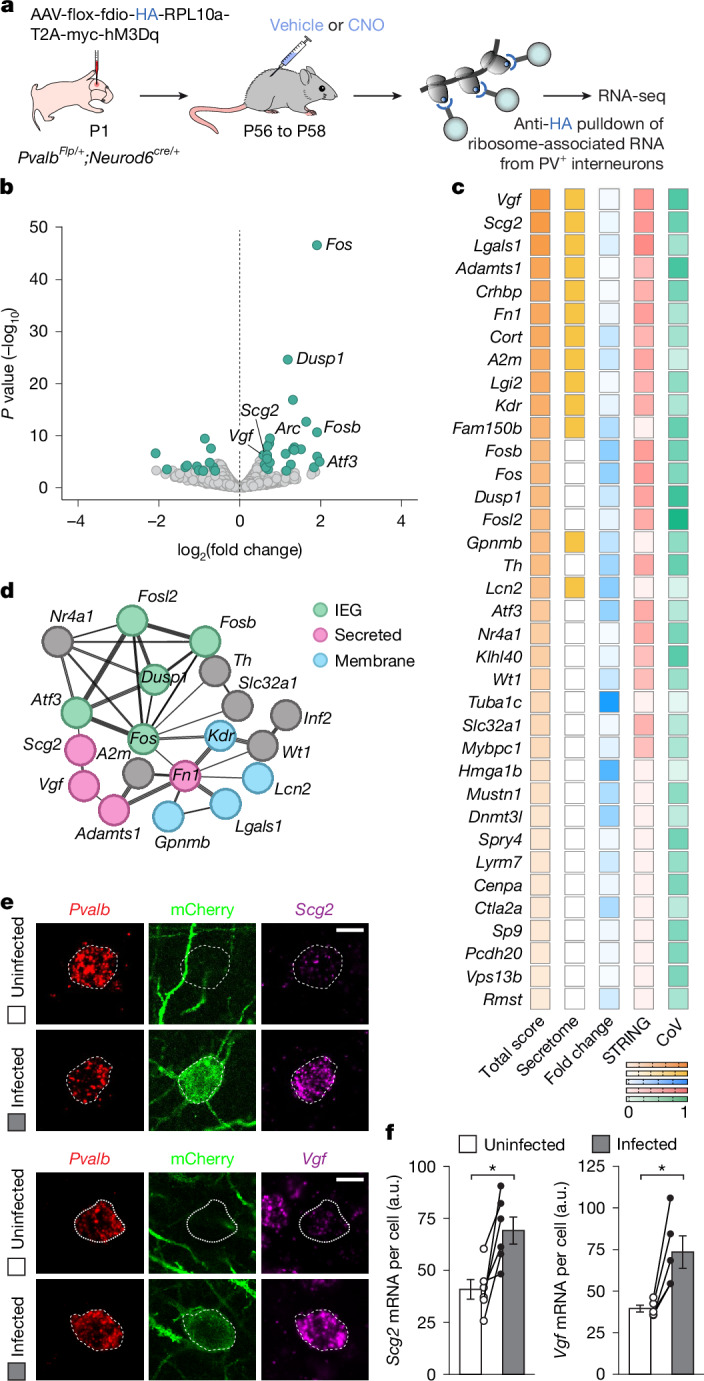
Increased activity in PV^+^ interneurons leads to upregulation of *Scg2* and *Vgf.* **a**, Experimental strategy. Adapted from ref. ^68^ (reprinted with permission from AAAS) and ref. ^69^. **b**, Volcano plot showing ribosome-associated mRNAs identified through RNA-seq. Differentially expressed RNAs (fold change > 1.5, *P* < 0.05, two-sided Wald’s test with adjustment for multiple testing) are labelled in teal. **c**, Ranking of the top DEGs on the basis of four selection criteria (Methods). Darker shades indicate higher scores (values 0 to 1). **d**, Partial STRING network of the most prominent node of DEGs, highlighting IEGs, and genes encoding secreted and membrane-bound proteins. **e**, *Scg2* and *Vgf* expression in neighbouring hM3Dq^+^ and uninfected PV^+^ interneurons in layer 2/3 of S1 from CNO-treated mice. Scale bar, 10 µm. **f**, Expression of *Scg2* (*n* = 6 mice; two-tailed paired Student’s *t*-test, *P* = 0.01) and *Vgf* (*n* = 5 mice; two-tailed paired Student’s *t*-test, *P* = 0.01) in neighbouring hM3Dq^+^ and uninfected PV^+^ interneurons from CNO-treated mice. Data are mean ± s.e.m. CoV, coefficient of variation. Source data

To identify the molecular programme through which PV^+^ interneurons modulate the input that they receive from other PV^+^ interneurons in response to changes in their activity, we set out to obtain the translatome of hM3Dq^+^ PV^+^ interneurons from control and CNO-treated mice. To this end, we used a viral approach to access ribosome-associated mRNAs by translating ribosome affinity purification^38^. In brief, we engineered Flp-dependent AAVs driving the bicistronic expression of haemagglutinin (HA)-tagged ribosomal protein (RPL10A) and MYC-tagged hM3Dq. Because PV is transiently expressed in some layer 5 pyramidal cells, we also flanked this construct with *LoxP* sites so that Cre-mediated recombination would prevent its expression. We injected this virus into S1 of *Pvalb*^*Flp/+*^*;Neurod6*^*cre/+*^ mice to achieve specific and sparse expression in PV^+^ interneurons (Extended Data Fig. 6h,i) and verified its effectiveness to activate hM3Dq^+^ PV^+^ interneurons using FOS (Extended Data Fig. 6j,k). We then isolated S1 from mice treated with vehicle or CNO for 48 h, pulled-down ribosomes using anti-HA beads to isolate ribosome-associated mRNA transcripts from PV^+^ interneurons, and quantified expression levels by RNA sequencing (RNA-seq) (Fig. 3a).

**Extended Data Fig. 7:**
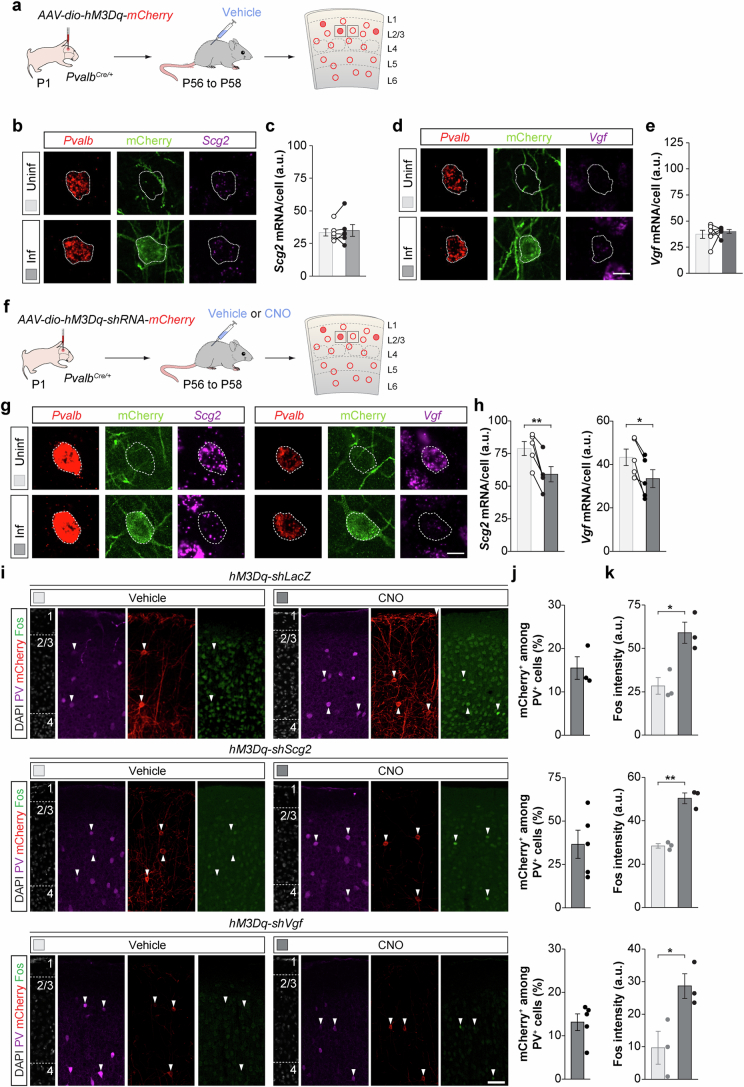
*Scg2* and *Vgf* expression following manipulations. **a**, Experimental strategy. **b**, Confocal images showing *Scg2* expression in in neighbouring infected and uninfected PV+ interneurons in vehicle-treated mice. **c**, Quantification of showing *Scg2* expression in in neighbouring infected and uninfected PV+ interneurons in vehicle-treated mice (*n* = 6 mice; two-tailed paired Student’s t-test, *p* = 0.54). **d**, Confocal images showing *Vgf* expression in in neighbouring infected and uninfected PV+ interneurons in vehicle-treated mice. **e**, Quantification of showing *Vgf* expression in in neighbouring infected and uninfected PV+ interneurons in vehicle-treated mice (*n* = 5 mice; two-tailed paired Student’s t-test, *p* = 0.58). **f**, Experimental strategy. **g**, *Scg2* and *Vgf* mRNA expression in neighbouring infected and uninfected PV^+^ interneurons in CNO-treated mice. **h**, Quantification of *Scg2* and *Vgf* mRNAs (*shScg2-mCherry*: *n* = 6 mice; two-tailed paired Student’s t-test, *p* = 0.007; *shVgf-mCherry*: *n* = 6 mice; two-tailed paired Student’s t-test, *p* = 0.02). **i**, Fos, mCherry and PV expression in coronal sections through S1 from vehicle- and CNO-treated mice infected with *LacZ-mCherry*, *shScg2-mCherry*, and *Vgf-mCherry*. **j**, Quantification of the percentage of mCherry^+^ PV^+^ interneurons in layer 2/3 of mice infected with *LacZ-mCherry* (*n* = 3 mice), *shScg2-mCherry* (*n* = 5 mice), and *Vgf-mCherry* (*n* = 5 mice). **k**, Quantification of the intensity of Fos staining in PV^+^ interneurons in vehicle- and CNO-treated mice infected with *LacZ-mCherry*, *shScg2-mCherry*, and *Vgf-mCherry* (*shLacZ-mCherry*: vehicle, *n* = 3 mice; CNO, *n* = 3 mice; two-tailed Student’s t-test, *p* = 0.02; *shScg2-mCherry*: vehicle, *n* = 3 mice; CNO, *n* = 3 mice; two-tailed Student’s t-test, *p* = 0.001; *Vgf-mCherry*: vehicle, *n* = 3 mice; CNO, *n* = 3 mice; two-tailed Student’s t-test, *p* = 0.04). Data are presented as mean ± s.e.m. Scale bars, 10 µm (b,c and g) and 50 µm (i). Schematics in **a**,**f** adapted from ref. ^68^ (reprinted with permission from AAAS) and ref. ^69^. Source data

We found 51 differentially expressed genes (DEGs), 37 upregulated and 14 downregulated, in PV^+^ interneurons from vehicle- and CNO-treated mice (Fig. 3b). Among the upregulated genes, we identified several immediate early genes (IEGs), such as *Fos*, *Fosb* and *Fosl2*, which is consistent with the increased activity of hM3Dq^+^ PV^+^ interneurons treated with CNO. We ranked DEGs according to several criteria, including fold expression change, biological reproducibility, the strength of predicted protein–protein interactions, and the secretory nature of the proteins (Fig. 3c, Extended Data Table 1 and Methods). The top candidates on this prioritized list included *Adamts1*, which encodes a disintegrin and metalloproteinase with thrombospondin motif protein family member responsible for cleaving lecticans in the perineuronal nets surrounding PV^+^ interneurons^39^, *Lgals1*, which encodes a glycoprotein of the galectin family developmentally enriched in PV^+^ basket cells^40^, and two neuropeptide-encoding genes, secretogranin II (*Scg2*) and *Vgf*, which have been linked to synaptic plasticity^18,41^. Protein–protein interaction network analysis by STRING^42^ revealed that many DEGs constitute an active node enriched in IEGs and other transcription factors, along with the two top candidates, *Scg2* and *Vgf* (Fig. 3d). We confirmed that *Scg2* and *Vgf* mRNA expression was upregulated following the activation of PV^+^ interneurons in vivo using single-molecule fluorescence in situ hybridization (Fig. 3e,f and Extended Data Fig. 7a–e). Together, these results revealed that cortical PV^+^ interneurons react to a sustained increase in their activity by inducing the expression of a molecular programme that includes IEGs, genes linked to the remodelling of perineuronal nets, and genes encoding secretable neuropeptides.

## Vgf mediates inhibitory plasticity in PV^+^ interneurons

To test whether *Scg2* and *Vgf* mediate the changes in PV–PV synaptic connectivity during plasticity, we sought to downregulate the expression of these genes, specifically in activated PV^+^ interneurons. To this end, we designed Cre-dependent AAVs expressing both hM3Dq and a short hairpin RNA (shRNA) targeting the candidate genes (sh*Scg2,* sh*Vgf* and shLacZ as a control) and validated their ability to downregulate the expression of the target genes (Extended Data Fig. 7f–h). We then performed three separate experiments injecting *Pvalb*^*cre/+*^ mice with AAVs conditionally expressing hM3Dq and shRNAs targeting *Scg2* (sh*Scg2-*hM3Dq), *Vgf* (sh*Vgf-*hM3Dq) or *LacZ* (sh*LacZ-*hM3Dq). As in previous experiments, viral injections were titrated to infect only a small population of PV^+^ interneurons. We also confirmed that the three vectors led to the activation of infected PV^+^ interneurons following CNO administration (Extended Data Fig. 7i–k).

**Fig. 4:**
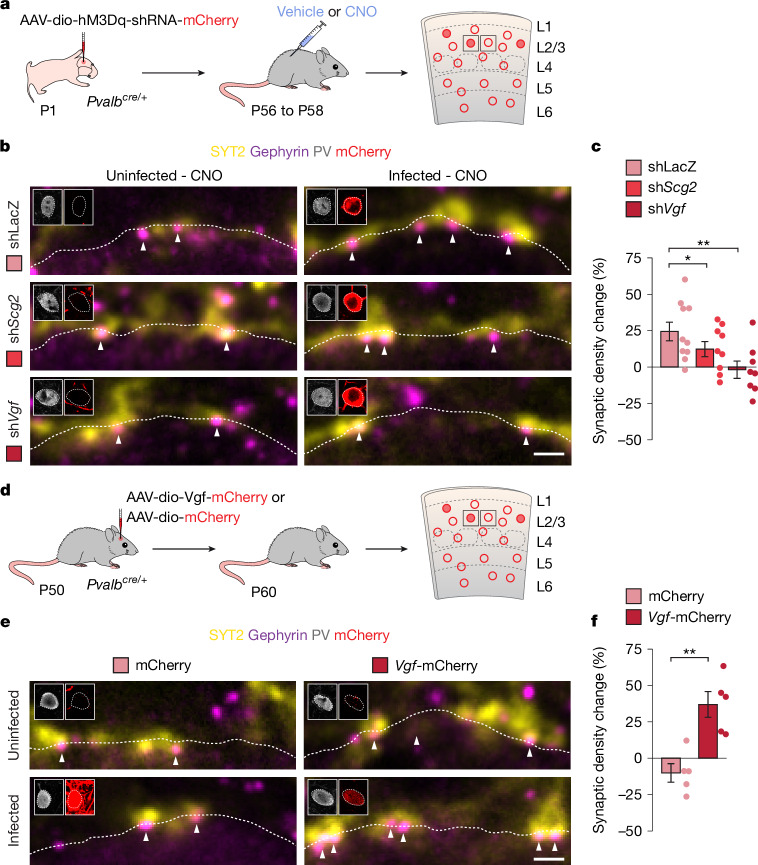
VGF is necessary and sufficient for scaling PV–PV connectivity. **a**, Experimental strategy. **b**, Presynaptic SYT2^+^ puncta and postsynaptic gephyrin^+^ clusters in shLacZ, sh*Scg2* and sh*Vgf* expressing PV^+^ interneurons in vehicle- and CNO-treated mice. Insets show PV and mCherry immunoreactivity in cell somata. Scale bar, 1 µm. **c**, Quantification of the change in synaptic density between infected and uninfected PV^+^ interneurons in CNO-treated mice injected with hM3Dq-shLacZ (*n* = 10 mice), hM3Dq-sh*Scg2* (*n* = 9 mice, two-tailed one-sample *t*-test, *P* = 0.049) and hM3Dq-sh*Vgf* (*n* = 8 mice, two-tailed one-sample *t*-test, *P* = 0.003). **d**, Experimental strategy. **e**, Presynaptic SYT2^+^ puncta and postsynaptic gephyrin^+^ clusters in mCherry- and *Vgf*-mCherry-expressing PV^+^ interneurons. Insets show PV and mCherry immunoreactivity in cell somata. Scale bar, 1 µm. **f**, Quantification of change in synaptic density between neighbouring uninfected and infected PV^+^ interneurons in mice injected with *Vgf-*mCherry (uninfected, *n* = 5 mice; CNO, *n* = 5 mice; two-tailed Student’s t-*t*est, *P* = 0.003). Data are mean ± s.e.m. Schematics in **a**,**d** adapted from ref. ^68^ (reprinted with permission from AAAS) and ref. ^69^. Source data

**Extended Data Fig. 8:**
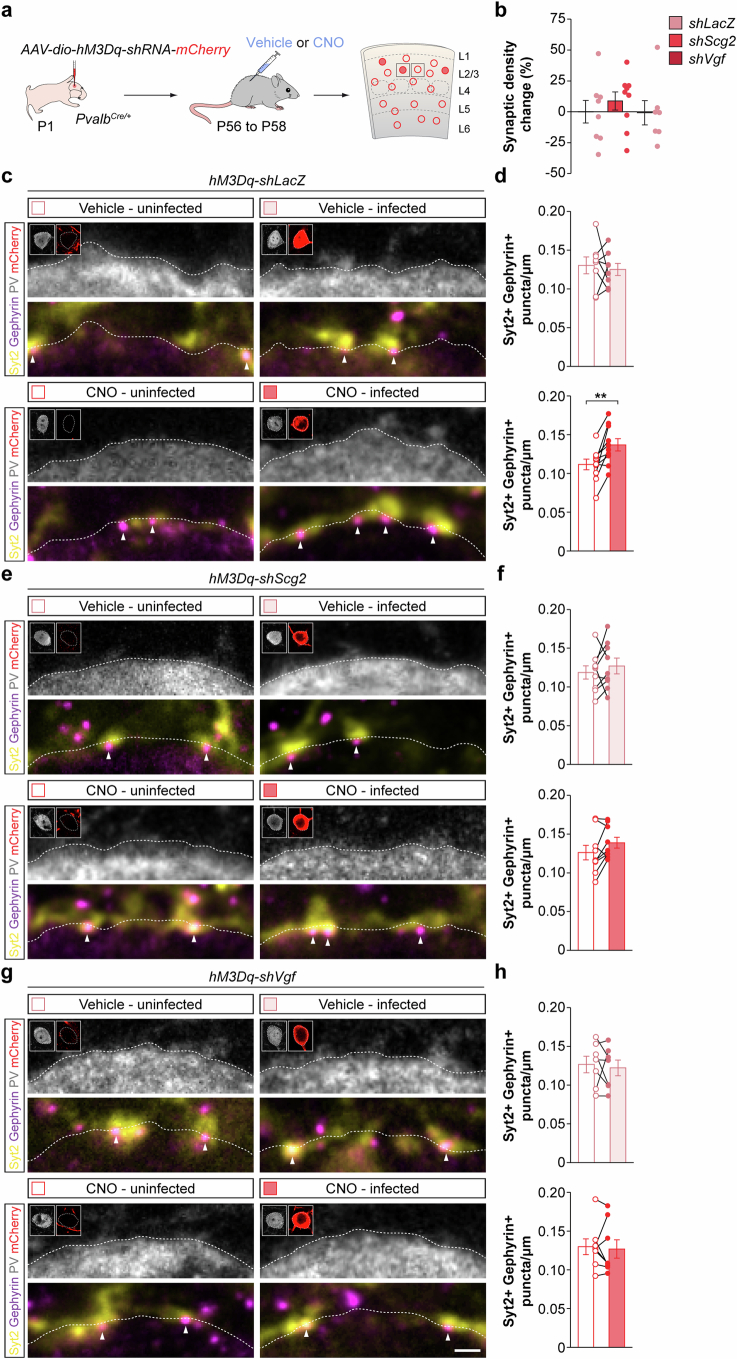
Downregulation of *Scg2* and *Vgf* prevents the increase in synaptic density induced by increased activity. **a**, Experimental strategy. Adapted from ref. ^68^ (reprinted with permission from AAAS) and ref. ^69^. **b**, Quantification of the change in synaptic density between infected and uninfected PV+ interneurons in vehicle-treated mice injected with *hM3Dq-shLacZ* (*n* = 8 mice), *hM3Dq-shScg2* (*n* = 9 mice, two-tailed one-sample t-test, *p* = 0.27) and *hM3Dq-shVgf* (*n* = 7 mice, two-tailed one-sample t-test, *p* = 0.94). **c**, Presynaptic Syt2^+^ puncta and postsynaptic Gephyrin^+^ clusters in neighbouring uninfected and infected PV^+^ interneurons in vehicle- and CNO-treated mice injected with *shLacZ-mCherry*. Inserts show PV and mCherry immunoreactivity in the cell somata. **d**, Quantification of the density of colocalising Syt2^+^/Gephyrin^+^ puncta on neighbouring uninfected and infected PV^+^ interneurons in vehicle- and CNO-treated mice injected with *shLacZ-mCherry* (vehicle: *n* = 8; two-tailed paired Student’s t-test, *p* = 0.67; CNO: *n* = 10; two-tailed paired Student’s t-test, *p* = 0.003). **e**, Presynaptic Syt2^+^puncta and postsynaptic Gephyrin^+^ clusters in neighbouring uninfected and infected PV^+^ interneurons in vehicle- and CNO-treated mice injected with *shScg2-mCherry*. Inserts show PV and mCherry immunoreactivity in the cell somata. **f**, Quantification of the density of colocalising Syt2^+^/Gephyrin^+^ puncta on neighbouring uninfected and infected PV^+^ interneurons in vehicle- and CNO-treated mice injected with *shScg2-mCherry* (vehicle: *n* = 9; two-tailed paired Student’s t-test, *p* = 0.40; CNO: *n* = 9; two-tailed paired Student’s t-test, *p* = 0.07). **g**, Presynaptic Syt2^+^ puncta and postsynaptic Gephyrin^+^ clusters in neighbouring uninfected and infected PV^+^ interneurons in vehicle- and CNO-treated mice injected with *shVgf-mCherry*. Inserts show PV and mCherry immunoreactivity in the cell somata. **h**, Quantification of the density of colocalising Syt2^+^/Gephyrin^+^ puncta on neighbouring uninfected and infected PV^+^ interneurons in vehicle- and CNO-treated mice injected with *shVgf -mCherry* (vehicle: *n* = 7; two-tailed paired Student’s t-test, *p* = 0.70; CNO: *n* = 8; two-tailed paired Student’s t-test, *p* = 0.72). Data are presented as mean ± s.e.m. Scale bar, 1 µm. Source data

We assessed the effect of downregulating *Scg2* or *Vgf* expression on the formation of PV^+^ synapses contacting hM3Dq^+^ PV^+^ interneurons following treatment with vehicle or CNO (Fig. 4a). We found no changes in the density of PV^+^ (SYT2^+^ gephyrin^+^) synapses contacting the soma of neighbouring infected and uninfected PV^+^ interneurons in vehicle-treated mice (Extended Data Fig. 8a). This result suggested that the prolonged downregulation of *Scg2* and *Vgf* alone does not modify the basal inhibitory connectivity of PV^+^ interneurons. We then examined whether the downregulation of *Scg2* or *Vgf* would interfere with the plasticity of inhibitory synapses contacting PV^+^ interneurons in CNO-treated mice. As expected, we observed a substantial increase in the density of SYT2^+^ gephyrin^+^ puncta contacting infected PV^+^ interneurons in mice injected with shLacZ-hM3Dq and treated with CNO (Fig. 4b,c). This effect was attenuated in mice injected with sh*Scg2-*hM3Dq and abolished in mice injected with sh*Vgf-*hM3Dq (Fig. 4b,c and Extended Data Fig. 8c–h). In other words, we continued to observe an increase in PV^+^ synapse density on PV^+^ interneurons infected with shLacZ following CNO treatment and PV^+^ interneurons infected with sh*Scg2* had a reduced ability to increase the number of PV^+^ synapses they received in response to an increase in their activity, whereas PV^+^ cells infected with sh*Vgf* were completely unable to increase the number of PV^+^ synapses they receive. These experiments demonstrated that the upregulation of *Scg2* and, most notably, *Vgf* in PV^+^ interneurons following their activation is required to form or stabilize synapses from other PV^+^ interneurons in response to changes in their activity.

**Extended Data Fig. 9:**
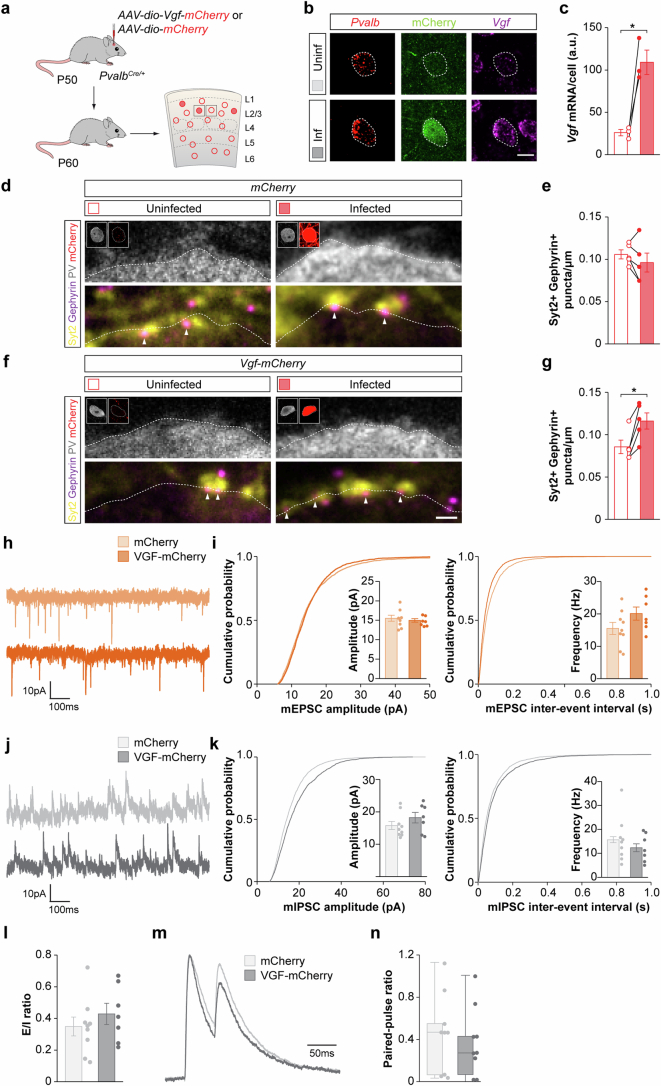
Overexpression of *Vgf* increases PV-PV interconnectivity but does not affect synaptic input or inhibitory release probability of PV^+^ interneurons. **a**, Experimental strategy. Adapted from ref. ^68^ (reprinted with permission from AAAS) and ref. ^69^. **b**, *Vgf* mRNA expression in neighbouring uninfected and Vgf-mCherry^+^ PV^+^ interneurons. **c**, Quantification of *Vgf* mRNA expression (*n* = 3 mice; two-tailed paired Student’s t-test, *p* = 0.03). **d**, Presynaptic Syt2^+^ puncta and postsynaptic Gephyrin^+^ clusters in neighbouring uninfected and infected mCherry^+^ PV^+^ interneurons. Inserts show PV and mCherry immunoreactivity in the cell somata. **e**, Quantification of the density of colocalising Syt2^+^/Gephyrin^+^ puncta on neighbouring uninfected and mCherry^+^ PV^+^ interneurons (*n* = 5; two-tailed paired Student’s t-test, *p* = 0.23). **f**, Presynaptic Syt2^+^ puncta and postsynaptic Gephyrin^+^ clusters in neighbouring uninfected and Vgf-mCherry^+^ PV^+^ interneurons. Inserts show PV and mCherry immunoreactivity in the cell somata. **g**, Quantification of the density of colocalising Syt2^+^/Gephyrin^+^ puncta on neighbouring uninfected and Vgf-mCherry^+^ PV^+^ interneurons (*n* = 5; two-tailed paired Student’s t-test, *p* = 0.01). **h**, Traces of mEPSCs recorded from mCherry expressing PV^+^ interneurons and VGF-mCherry expressing PV^+^ interneurons. **i**, Quantification of amplitude (mCherry, *n* = 9 cells, 9 slices, 4 mice; VGF-mCherry, *n* = 7 cells, 7 slices, 4 mice; two-tailed Student’s t-test, *p* = 0.59) and frequency (mCherry, *n* = 9 cells, 9 slices, 4 mice; VGF-mCherry, *n* = 7 cells, 7 slices, 4 mice; two-tailed Student’s t-test, *p* = 0.12) of mEPSCs recorded from mCherry expressing PV+ interneurons and VGF-mCherry expressing PV^+^ interneurons. **j**, Traces of mIPSCs recorded from mCherry expressing PV^+^ interneurons and VGF-mCherry expressing PV^+^ interneurons. **k**, Quantification of amplitude (mCherry, *n* = 9 cells, 9 slices, 4 mice; VGF-mCherry, *n* = 7 cells, 7 slices, 4 mice; two-tailed Student’s t-test, *p* = 0.26) and frequency (mCherry, *n* = 9 cells, 9 slices, 4 mice; VGF-mCherry, *n* = 7 cells, 7 slices, 4 mice; two-tailed Student’s t-test, *p* = 0.42) of mIPSCs recorded from mCherry expressing PV^+^ interneurons and VGF-mCherry expressing PV^+^ interneurons. **l**, Excitation/Inhibition (E/I) ratio in mCherry expressing PV^+^ interneurons and VGF-mCherry expressing PV^+^ interneurons (mCherry, *n* = 9 cells, 9 slices, 4 mice; VGF-mCherry, *n* = 7 cells, 7 slices, 4 mice; two-tailed Student’s t-test, *p* = 0.38). **m**, Traces of inhibitory pair pulses recorded from mCherry expressing PV^+^ interneurons and VGF-mCherry expressing PV^+^ interneurons with a 50 ms inter-stimulus interval. **n**, Quantification of paired-pulse ratio (mCherry, *n* = 9 cells, 9 slices, 4 mice; VGF-mCherry, *n* = 10 cells, 10 slices, 4 mice; two-tailed Student’s t-test, *p* = 0.46). Bar graphs are presented as mean ± s.e.m. Box plot centre line indicates the median, box limits indicate upper and lower quartiles, whiskers indicate maximum and minimum. Source data

We then tested whether increased expression of *Vgf* is sufficient for the increase in PV–PV connectivity in the absence of increased activity. To this end, we generated Cre-dependent AAVs to overexpress VGF–mCherry or mCherry alone, injected them into S1 of postnatal day 50 (P50) *Pvalb*^*cre/+*^mice, and assessed changes in inhibitory synaptic density ten days after injection (Fig. 4d and Extended Data Fig. 9a–c). We found a significant increase in the density of PV^+^ synapses targeting PV^+^ interneurons expressing VGF–mCherry compared with uninfected neighbouring control cells (Fig. 4e,f and Extended Data Fig. 9f,g). By contrast, we did not observe this increase in PV^+^ interneurons infected with mCherry alone (Fig. 4e,f and Extended Data Fig. 9d,e). To test whether the increase in structural PV–PV connectivity resulted in altered synaptic transmission, we recorded mEPSCs and mIPSCs from PV^+^ interneurons expressing VGF–mCherry or mCherry alone. We found no change in the amplitude and frequency of mEPSCs or mIPSCs (Extended Data Fig. 9h–l). In addition, we found no changes in the inhibitory paired-pulse ratio (Extended Data Fig. 9m,n), indicating that overexpression of VGF does not affect release probability. These experiments demonstrate that increased VGF expression, without increased activity, is sufficient to increase structural connectivity among PV interneurons. However, additional activity-dependent factors seem required for the functional plasticity of these connections.

## Network activation increases VGF expression and PV–PV connectivity

**Extended Data Fig. 10:**
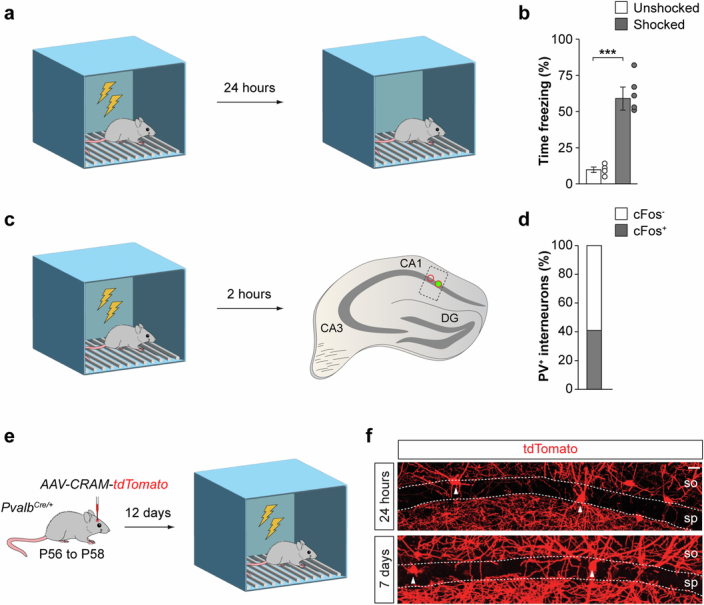
Contextual fear conditioning activates a sparse population of PV^+^ interneurons. **a**, Experimental strategy. **b**, Quantification of the percentage of time spent freezing for control (unshocked) and previously shocked mice during re-exposure to the cFC environment after 24 h (unshocked, *n* = 4 mice; shocked, *n* = 5 mice; two-tailed Student’s t-test, *p* = 8 × 10^−5^). **c**, Experimental strategy. **d**, Quantification of the number of Fos^+^ PV^+^ interneurons 2 h after cFC (*n* = 117 cells). **e**, Experimental strategy. **f**, Example images of tdTomato+ cells 1 (6 mice) and 7 (7 mice) days after cFC. Data are presented as mean ± s.e.m. Scale bar, 25 µm (f). Schematics in **a**,**c**,**e** adapted from ref. ^69^. Source data

Chemogenetic activation of a small subset of PV^+^ interneurons leads to the expression of VGF and other factors in the activated cells and a subsequent increase in PV–PV connectivity. To explore whether this regulation of PV–PV connectivity also occurs in physiologically relevant conditions, we used contextual fear conditioning (cFC), an experimental model of fear learning. This single-trial paradigm increases the activity of a sparse population of pyramidal cells and PV^+^ interneurons in the CA1 region of the hippocampus, which are thought to encode a memory trace^43,44^. We confirmed that mice receiving three foot shocks displayed significantly longer freezing when re-exposed to the experimental environment than control unshocked mice, demonstrating the successful association of foot shocks to the experimental environment (Extended Data Fig. 10a,b). In these mice, a fraction of CA1 PV^+^ interneurons expresses FOS 2 h after cFC (Extended Data Fig. 10c,d), confirming that this behavioural paradigm reliably activates a subset of PV^+^ interneurons in a time-locked manner.

**Fig. 5:**
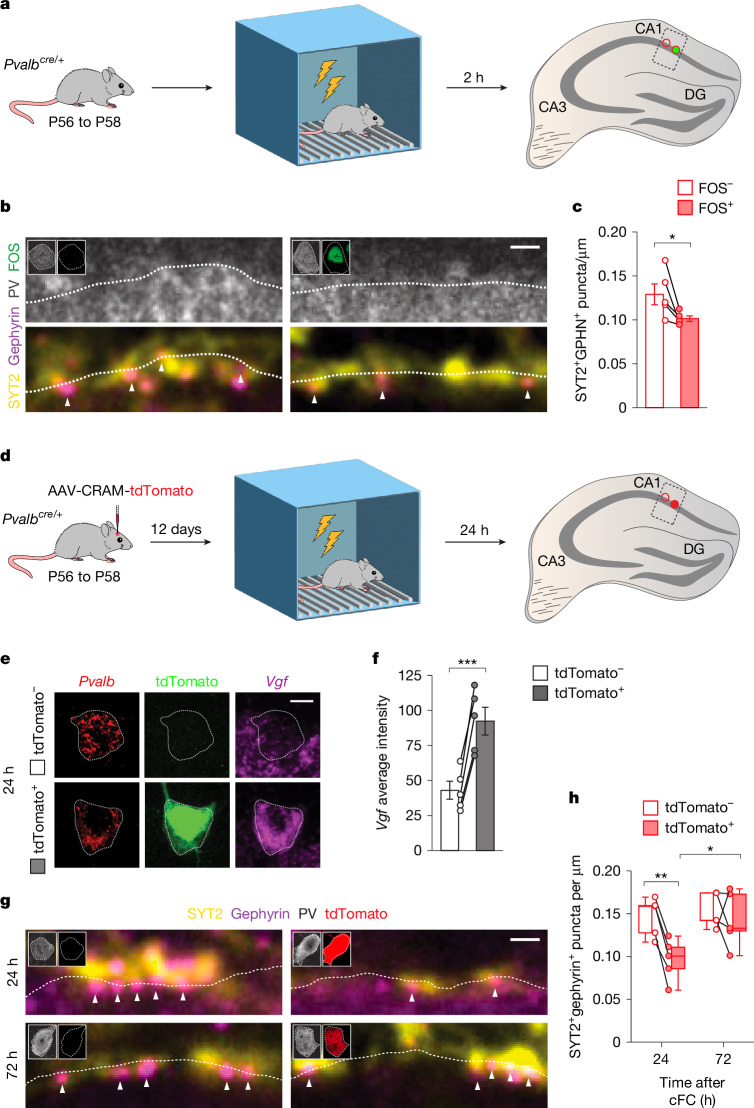
Contextual fear conditioning increases *Vgf* expression and PV–PV connectivity in activated PV^+^ interneurons. **a**, Experimental strategy. **b**, Presynaptic SYT2^+^ puncta and postsynaptic gephyrin^+^ clusters on FOS^−^ and FOS^+^ PV^+^ cells 2 h after cFC. Insets show PV and FOS immunoreactivity in cell somata. **c**, Quantification of PV^+^ synaptic density onto FOS^−^ and FOS^+^ PV^+^ interneurons 2 h after cFC (FOS^−^, *n* = 5 mice; FOS^+^, *n* = 5 mice; two-tailed Student’s *t*-test, *P* = 0.044). **d**, Experimental strategy. **e**, *Vgf* expression 1 h after cFC in activated (tdTomato^+^) and neighbouring, non-activated (tdTomato^−^) PV^+^ interneurons. **f**, Quantification of *Vgf* intensity in tdTomato^−^ and tdTomato^+^ PV interneurons (tdTomato^−^, *n* = 5 mice; tdTomato^+^, *n* = 5 mice; two-tailed paired Student’s *t*-test, *P* = 8 × 10^-4^). **g**, Presynaptic SYT2^+^ puncta and postsynaptic gephyrin^+^ clusters in tdTomato^−^ and tdTomato^+^ PV^+^ interneurons 24 and 72 h after cFC. **h**, Quantification of the change in synaptic density between activated (tdTomato^+^) and non-activated (tdTomato^−^) PV^+^ interneurons 24 and 72 h after cFC. 24 h: *n* = 5 mice; 72 h: *n* = 5. tdTomato^−^ versus tdTomato^+^ at 24 h: two-tailed paired Student’s *t*-test, *P* = 0.001; tdTomato^+^ at 24 h versus tdTomato^+^ at 72 h: two-tailed Student’s *t*-test, *P* = 0.033. Data are presented as mean ± s.e.m. In box plots, the centre line indicates the median, box limits delineate middle quartiles, and whiskers indicate maximum and minimum. Scale bars: 10 µm (**e**) and 1 µm (**b**,**g**). Schematics in **a**,**d** adapted from ref. ^69^. Source data

Because the inhibition received by PV^+^ interneurons may factor in their recruitment in response to cFC, we first assessed PV–PV connectivity to determine the baseline of activated PV^+^ interneurons in these experiments. To this end, we quantified the density of PV^+^ (SYT2^+^ gephyrin^+^) synapses onto activated (FOS^+^) and neighbouring, non-activated (FOS^−^) PV^+^ interneurons 2 h after cFC (Fig. 5a) when FOS expression peaks, but before there is time for structural changes in inhibitory synapses^45,46^. We found that FOS^+^ PV^+^ interneurons consistently received fewer synapses from other PV^+^ cells than neighbouring FOS^−^ PV^+^ cells (Fig. 5b,c). This observation revealed that cFC preferentially recruits CA1 PV^+^ interneurons among those receiving less inhibition from other PV^+^ cells.

We next examined whether the activation of PV^+^ interneurons through cFC leads to increased *Vgf* expression and PV–PV connectivity, as observed in the chemogenetic experiments. Since FOS is only transiently expressed following increased neuronal activity^45,46^, we used Cre-dependent robust activity marking (CRAM)^47^ to label the cFC-activated PV^+^ interneurons beyond the time of FOS expression. CRAM leads to the Cre-dependent expression of tdTomato from an activity-dependent promotor in a Tet^off^ system, allowing the persistent labelling of cells expressing FOS in a time-locked manner (that is, following induction by cFC). When performing these experiments in *Pvalb*^*cre/+*^ mice, CRAM consistently labelled a small subset of PV^+^ interneurons up to seven days after cFC (Extended Data Fig. 10e,f), thereby distinguishing cFC-activated (tdTomato^+^) and neighbouring non-activated (tdTomato^−^) PV^+^ interneurons.

To examine whether cFC led to a sustained increase in *Vgf* expression, we injected CRAM in the hippocampus of *Pvalb*^*cre/+*^ mice, performed cFC, and analysed the brains 24 h later (Fig. 5d). We observed that *Vgf* expression was increased in activated tdTomato^+^ PV^+^ cells compared with neighbouring, non-activated tdTomato^−^ PV^+^ interneurons (Fig. 5e,f), demonstrating that network-driven activation of PV^+^ interneurons through a behavioural paradigm increases *Vgf* expression in these cells.

Next, we examined whether the *Vgf* expression induced by cFC is followed by an increase in PV–PV connectivity, as observed in our chemogenetic experiments. Consistent with our observation in FOS^+^ cells (Fig. 5b,c), we found that activated tdTomato^+^ PV^+^ interneurons received a markedly lower (around 34%) density of PV^+^ synapses than neighbouring non-activated PV^+^ cells 24 h after cFC (Fig. 5g,h). However, the density of inhibitory synapses received by activated tdTomato^+^ PV^+^ markedly increased over the following 48 h, whereas no significant changes were observed in neighbouring non-activated PV^+^ cells (Fig. 5g,h). In sum, these experiments revealed that cFC preferentially recruits PV^+^ interneurons with low PV–PV connectivity and induces *Vgf* expression in these cells, which progressively increases the inhibition they receive from other PV^+^ cells over the next few days.

## Discussion

Our results shed light on a fundamental process that mediates the efficient balance of excitation and inhibition in the cerebral cortex. Pyramidal cells balance the excitation they receive by dynamically adjusting their inhibitory inputs, specifically from PV^+^ interneurons^16^. We demonstrate here that PV^+^ interneurons are also responsible for regulating the activity of other PV^+^ interneurons through the modulation of their inhibitory synapses. Connections among cortical PV^+^ interneurons are more frequent than among any other type of interneuron or between other interneurons and PV^+^ interneurons^48–52^. PV^+^ interneurons also frequently exhibit autapses—synapses that a neuron makes onto itself—that adjust their temporal firing interval with extreme accuracy^53,54^. In addition to chemical synapses, PV^+^ interneurons are frequently interconnected through gap junctions^49,55^. Electrical synapses enhance synchrony between PV^+^ interneurons^56^, but their function in activity-dependent plasticity remains to be established. The dynamic regulation of the connectivity among PV^+^ interneurons is likely to influence their firing rate, the modulation of gamma rhythms^57,58^ and, as shown by our experiments, their recruitment into engrams, processes that are essential for sensory perception, attention, learning and memory. Our results also suggest that changes in PV^+^ interconnectivity may contribute to consolidating neuronal ensembles that encode experiences.

The activity-dependent increase in PV–PV connectivity following increased activity involves changes in gene expression, a process that is likely to be coordinated by members of the FOS family of transcription factors^59^. In the hippocampus, FOS-dependent changes in the expression of *Scg2* in pyramidal cells modulate the inhibition these cells received from PV^+^ interneurons^18^. Our study shows that VGF critically regulates PV–PV connectivity in response to increased activity. However, whereas VGF expression increases structural synaptic connectivity, other activity-dependent molecules, such as Scg2, might regulate synaptic strength. Consistent with that, ionotropic GABA-A receptors are dynamically inserted into inhibitory synapses in a homeostatic manner, and structural synapses lacking GABA receptors have been observed following synaptic downscaling^60^. These observations suggest that neuropeptide-encoding genes may have evolved to modulate the activity-dependent plasticity of different components of inhibitory connectivity in the cerebral cortex.

VGF is a neuropeptide precursor of the extended granin family. These proteins are stored and sorted into dense core vesicles, proteolytically processed and secreted as small bioactive peptides^61^. Several secreted neuropeptides derived from VGF, including TLQP-62 and TLQP-21, have been linked to memory formation in the hippocampus and energy homeostasis in the hypothalamus^62,63^, although their mechanism of action remains unclear. Determining the specific VGF-derived peptides involved in synapse formation, their presynaptic and postsynaptic sites of action, and the putative G-protein-coupled receptor mediating their function in PV^+^ interneurons will be critical for establishing the precise mechanisms through which VGF regulates inhibitory synaptic plasticity in these cells.

Dysregulation of VGF expression has been associated with multiple neurodegenerative and psychiatric conditions, most notably in Alzheimer’s disease and mood disorders^64^. Alterations in PV^+^ interneurons may mediate functional deficits in many conditions^65–67^. Identifying a critical role for VGF in regulating synaptic connectivity among PV^+^ interneurons provides a path to mechanistically understand its contribution to the plasticity of cortical circuits in health and disease.

## Methods

### Mice

#### Mouse lines and breeding

All experiments followed King’s College London Biological Service Unit’s guidelines and the European Community Council Directive of 24 November 1986 (86/609/EEC). Animal work was carried out under licence from the UK Home Office following the Animals (Scientific Procedures) Act 1986. Similar numbers of male and female mice were used in all experiments. Animals were maintained under standard laboratory conditions on a 12:12 h light:dark cycle in individual ventilated cages at 22 ± 2 °C and 55 ± 10% relative humidity with water and food ad libitum. Sample sizes in animal experiments were chosen to match standards in the field. Experimental conditions were randomized or alternated within litters where possible. The *Pvalb*^*cre/+*^ mice used in most experiments were generated by crossing *Pvalb*^*cre/+*^ mice (*B6.129P2-Pvalb*^*tm1(cre)Arbr*^*/J*, JAX017320) with CD1 mice (Crl:CD1[ICR], Charles River). To generate *Pvalb*^*cre/Flp*^*;RCL*^*ChR2/+*^, *Sst*^*cre/+*^*;Pvalb*^*Flp/+*^*;RCL*^*ChR2/+*^ and *Vip*^*cre/+*^*;Pvalb*^*Flp/+*^*;RCL*^*ChR2/+*^ mice, we first generated *Pvalb*^*Flp/Flp*^*;RCL*^*ChR2/ChR2*^ mice by crossing *Pvalb*^*Flp/Flp*^ mice (*B6.Cg-Pvalb*^*tm4.1(flpo)Hze*^*/J*, JAX022730) with *RCL*^*ChR2/ChR2*^ (Ai32) mice (*B6;129S-Gt(ROSA)26Sor*^tm32(CAG-COP4*H134R/EYFP)Hze/^*J*, JAX012569). These mice were then crossed with *Pvalb*^*cre/cre*^, *Sst*^*cre/cre*^ (*Sst*^*tm2.1(cre)Zjh*^*/J*, JAX013044) or *Vip*^*cre/cre*^ (*Vip*^*tm1(cre)Zjh*^*/J*, JAX010908) mice. *Pvalb*^*Flp/+*^*;NeuroD6*^*cre/+*^ mice were generated by crossing *Pvalb*^*Flp/+*^ mice with *Nex*^*cre/+*^ (*Neurod6*^*tm1(cre)Kan*^) mice^70^.

#### Contextual fear conditioning

The day prior to conditioning, animals were briefly handled by the experimenter for 1–2 min. On the following day, mice were transferred to the experimental room in separate cages and allowed to acclimatize for 15–20 min before being placed in the experimental chamber (VFC-008 Fear Conditioning Chamber in a NIR-022SD Sound attenuating Cubicle, Med Associates). Mice received shocks following a standard protocol^71^. Animals were allowed to acclimatize for 3 min, followed by three mild foot shocks (2 s, 0.7 mA), with 30-s intervals between each shock. Post-shock freezing behaviour was measured for 30 s. The animals were returned to the holding cages and placed back into their home cages 15 min later. For the retrieval experiment, mice were returned to the experimental room in the same holding cages used the previous day. After a 15-min acclimatization period, they were placed in the experimental chamber, and freezing behaviour was recorded for 5 min. Freezing was defined as the absence of movement except for respiration and was measured and analysed using Video Freeze (Med Associated).

#### Doxycycline treatment and CRAM timeline

For CRAM experiments, mice were put on doxycycline-enriched diet (Envigo, TD.10483, 40 mg kg^−1^) immediately after surgery and left on this diet for a minimum of 10 days to allow for expression of the construct without expression of tdTomato. Forty-eight hours before cFC, mice were moved back to standard food to allow tdTomato expression following FOS expression upon cFC. In experiments lasting more than 48 h, mice were put back on doxycycline-enriched diet 48 h after cFC to prevent unspecific tdTomato expression.

#### CNO treatment

Mice between P42 and P70 were treated with vehicle or CNO (Tocris, 4936) as indicated. Mice used for FOS experiments received a single injection of vehicle or CNO (1 mg kg^−1^) and were perfused 2 h later. In all other experiments, mice were treated with vehicle or CNO (1 mg kg^−1^) for ~48 h via 5 injections. Injections were administered starting 2 days before the experimental day and took place in the morning (between 08:00 and 10:00) and in the evening (between 18:00 and 21:00). A fifth injection was administered between 08:00 and 10:00 on the day of the experiment. CNO was dissolved in 0.5% dimethyl sulfoxide (DMSO, Sigma, D0418) in 0.9% saline and stored at −20 °C.

### Histology

#### Immunohistochemistry

Mice were deeply anaesthetized with pentobarbital before being transcardially perfused with 0.9% saline, followed by 4% paraformaldehyde in PBS. Brains were post-fixed for 2 h in 4% paraformaldehyde at 4 °C, followed by cryoprotection in 15% and 30% sucrose. Brains were sectioned at 40 µm on a sliding microtome (Leica SM2010R) and stored in ethylene glycol (30% ethylene glycol (Merck, 324558), 30% Glycerol (Merck, G5516) in PBS) at −20 °C. Free-floating sections were washed in 0.25% Triton X-100 (Merck) in phosphate-buffered saline (PBS) before 2 h incubation in blocking buffer containing 0.25% Triton X-100, 10% serum and 2% bovine serum albumin in PBS. Sections were incubated overnight at 4 °C in a blocking buffer with primary antibodies. The next day, sections were washed in 0.25% Triton X-100 in PBS and incubated for 2 h in a blocking buffer with secondary antibodies at room temperature. Sections were washed in PBS and stained with 5 µM 4′,6-diamidino-2-phenylindole (DAPI) (Merck) in PBS if required. Sections were dried and mounted with Mowiol/DABCO (8% Mowiol (SLS, 81381), 2% Dabco (Merck, D27802) in PBS). The following primary antibodies and concentrations were used: goat anti-mCherry (1:500, Antibodies-Online, ABIN1440057), dsRed anti-rabbit (1:500, Clontech, 632496), FOS anti-rabbit (1:200, Merck, ABE457), gephyrin anti-mouse-IgG1 (1:500, Synaptic Systems, 147011), VGaT anti-guinea pig (1:500, Synaptic Systems, 131004), parvalbumin anti-chicken (1:250, Synaptic Systems, 195006), parvalbumin anti-mouse (1:3000, Swant, 235) and synaptotagmin-2 anti-mouse-IgG2 (1:250, ZFIN, ZDB-ATB-081002-25). The following secondary antibodies and concentrations were used: Donkey anti-Chicken 405 (1:200, Jackson, 703-475-155), Goat anti-Mouse-IgG1 488 (1:500, Molecular Probes, A21121), Donkey anti-Rabbit 488 (1:400, Thermo Fisher Scientific, A21206), Donkey anti-Rabbit 555 (1:400, Molecular Probes, A31572), Donkey anti-Goat 555 (1:400, Invitrogen, A21432), Goat anti-Mouse-IgG2 647 (1:500, Molecular Probes, A21241), Donkey anti-Guinea Pig 647 (1:250, Jackson, 706-605-148) and Donkey anti-Mouse IgG1 647 (1:400, Thermo Fisher Scientific, A31571).

#### Single-molecule fluorescence in situ hybridization

Brains were perfused, sectioned and stored in RNase-free solutions. Mice were perfused as described above and post-fixed for 24 h before cryoprotected in 15% and 30% sucrose. The brains were sectioned at 30 µm on a sliding microtome and stored at 20 °C. Sections were mounted on RNase-free SuperFrost Plus slides (Thermo Fisher) and probed following the RNAscope Multiplex Fluorescent Assay v2 protocol (ACDBio 323110). The following probes from ACDBio were used: Mm-Scg2-C1 (477691), Mm-Vgf-C1 (517421) and Mm-Pvalb-C2 (421931).

#### Image acquisition

Image acquisition and image analysis was performed blind to the treatment condition (CNO or vehicle) of the sample. Images were acquired using an inverted SP8 confocal microscope using the LAS AF software version 3.5.7.23225 (Leica). Different experimental conditions within an experiment were always imaged in parallel, and imaging settings were kept constant for each experiment. All images were taken at 200 Hz acquisition speed and 1,024 × 1,024-pixel resolution. Infection density and FOS intensity were imaged using a 10× objective (0.8800 pixels per µm). RNAscope images were acquired using a 63× objective (5.5440 pixels per µm). Synaptic quantification images were taken using a 100× objective at 1.75× digital zoom (15.4000 pixels per µm). Imaging was restricted to S1 layer 2/3 unless otherwise stated. To image neighbouring cells, we aimed to image an infected and an uninfected cell as close as possible together at the same distance from the pial surface (for example, after imaging an infected PV^+^ interneuron, an effort was made to image an uninfected PV^+^ interneuron nearby and at a similar depth from the pial surface).

#### Image analysis

FOS intensity was analysed using a custom MATLAB script. Cell bodies were segmented using disk morphological shape function, size, and intensity thresholding to create individual regions of interest (ROIs). Layer 2/3 was determined using DAPI counterstaining. ROIs in layer 2/3 were used to measure average signal intensity in the FOS channel.

For single-molecule fluorescence in situ hybridization experiments, the number of mRNA particles was determined using a custom MATLAB script. In brief, for each image, background subtraction was applied. The contour of *Pvalb*^+^ cells was then used to draw an ROI and labelled as mCherry^+^ or mCherry^−^ after visual inspection. Within each ROI, the total area of the signal was measured. This area is then divided by the area of a single mRNA particle (∼0.16 µm^2^), which estimates the number of mRNA particles in the ROI. We applied intensity correction to normalize signal intensity differences between brains. For this, we measured the average signal intensity of each image, excluding the ROI of the PV^+^ interneurons and calculated the average signal intensity for each brain. We normalized these values to the lowest value to get the intensity ratio. We then divided the number of mRNA particles by the intensity ratio.

Synaptic densities were analysed using a custom FIJI script, as previously described^32^. Background subtraction, Gaussian blurring, smoothing and contrast enhancement were applied in all channels. Cell somata were drawn automatically or manually on the basis of intensity levels of parvalbumin staining to create a mask of the soma surface and measure its perimeter. Presynaptic boutons and postsynaptic clusters were detected automatically on the basis of thresholds of intensity. Thresholds for the different synaptic markers were selected from a set of random images before quantification, and the same threshold was applied to all images from the same experiment. The ‘Analyze Particles’ and ‘Adjustable Watershed’ tools were applied to the synaptic channels, and a mask was generated with a minimum particle size of 0.05. The soma mask and the corresponding synaptic masks were merged to quantify the number of puncta contacting the soma and dendrite. Puncta were defined as presynaptic boutons when they were located outside the soma or dendrite and had ≥0.1 µm^2^ colocalizing with the soma or dendrite perimeter. Puncta were defined as postsynaptic clusters inside a soma or dendrite and had ≥0.2 µm^2^ colocalising with the soma or dendrite perimeter. Synapses were defined as presynaptic boutons and postsynaptic clusters contacting each other with a colocalization area of ≥0.03 µm^2^ of their corresponding masks. Classification of cells as uninfected or infected was performed manually on the basis of the absence or presence of mCherry signal, respectively. The density change was calculated as:

$${\rm{Density}}\;{\rm{change}}=\left(\frac{{\rm{Synapse}}\;{\rm{density}}\;{\rm{infected}}\;{\rm{cells}}}{{\rm{Synapse}}\;{\rm{density}}\;{\rm{uninfected}}\;{\rm{cells}}}-1\right)\times 100$$

### Viruses

#### Virus production

AAV8 viruses were produced in HEK293FT cells grown on 5 plates (linear PEI, Polysciences Europe, 23966-100) or 10 plates (branched PEI, Sigma-Aldrich, 408727) of 15 cm diameter until they reached 60% confluency. Cells were grown on DMEM (Gibco 21969-035) + 10% fetal bovine serum (FBS) (Gibco 10500-064) + 1% penicillin/streptomycin (Gibco 15140-122) and 10 mM HEPES. AAVs were produced using polyethylenimine (PEI) transfection of HEK293FT cells with a virus-specific transfer plasmid (70 µg per 10 plates) and a pDP8.ape helper plasmid (300 µg per 10 plates, PF478 from PlasmidFactory). The helper plasmid provided the AAV Rep and Cap functions and the Ad5 genes (VA RNAs, E2A and E4). The DNA and PEI were mixed in a 1:4 ratio in uncomplemented DMEM and left at room temp for 25 mins to form the DNA–PEI complex. The transfection solution was added to each plate and incubated for 72 h at 37 °C in 5% CO_2_. The transfected cells were then scraped off the plates and pelleted. The cell pellet was lysed in buffer containing 50 mM Tris-Cl, 150 mM NaCl, 2 mM MgCl_2_, and 0.5% sodium deoxycholate and incubated with 100 U ml^−1^ benzonase (Sigma, E1014 25KU) for 1 hr to dissociate particles from membranes. The particles were cleared by centrifugation, and the clear supernatant was filtered through 0.8 µm (Merck Millipore Ref SLAA033SS) and 0.45 µm (Merck Millipore Ref SLHA 033SS) filters. The viral suspension was loaded on a discontinuous iodixanol gradient using 4 layers of different iodixanol concentrations^72^ of 15, 25, 40 and 58% in Quick-seal polyallomer tubes (Beckman Coulter, 342414) and spun in a VTi-50 rotor at 50,000 rpm for 75 min at 12 °C in an Optima L-100 XP Beckman Coulter ultracentrifuge to remove any remaining contaminants. After centrifugation, 5 ml were withdrawn from the 40/58% interface using a G20 needle. The recovered virus fraction was purified by first passing through a 100-kDA molecular mass cut-off centrifugal filter (Sartorius VIVASPIN VS2041) and then through an Amicon Ultra 2 ml Centrifugal filter (Millipore UFC210024). Storage buffer (350 mM NaCl and 5% Sorbitol in PBS) was added to the purified virus, and 5 µl aliquots were stored at −80 °C.

The following viruses were used in this study: AAV8-hSyn-DIO-hM3D(Gq)-mCherry (Addgene 44361), AAV8-hSyn-DIO-mCherry (Addgene 50459), AAV8-hSyn-DIO-hM4D(Gi)-mCherry (Addgene 44362), AAV8-hSyn-fDIO-hM3D(Gq)-mCherry (Addgene 223652), AAV8-hSyn-flx-fDIO-HA-Rpl10a-T2A-Myc-hM3D(Gq) (Addgene 223654), AAV8-hSyn-DIO-hM3D(Gq)-mCherry-shLacZ-CWB (shRNA sequence: AAATCGCTGATTTGTGTAGTC) (Addgene 223660), AAV8-hSyn-DIO-hM3D(Gq)-mCherry-shScg2-CWB (shRNA sequence: GCAGACAAGCACCTTATGAA) (Addgene 223659), AAV8-hSyn-DIO-hM3D(Gq)-mCherry-shVgf-CWB (shRNA sequence: GACGATCGATAGTCTCATTGA) (Addgene 223658), AAV8-hSyn-DIO-Vgf-T2A-mCherry-CW3SL (Addgene 223661), AAV8-hSyn-DIO- mCherry-CW3SL (Addgene 223662), and AAV8-CRAM-tdTomato (Addgene 84468). AAV8-hSyn-DIO-hM3D(Gq)-mCherry, AAV8-hSyn-DIO-mCherry and AAV8-hSyn-DIO-hM4D(Gi)-mCherry were a gift from B. Roth^73^. pAAV-CWB-EGFP and pAAV-CW3SL-EGFP backbones were used to create plasmids as indicated and were a gift from B.-K. Kaang^74^. pAAV-hSyn-FLExFRT-mGFP-2A-Synaptophysin-mRuby was used to create fDIO plasmids and was a gift from L. Luo^75^. pAAV-CRAM-tdTomato was a gift from Y. Lin^47^. All viruses were diluted to a titre between 5 × 10^11^ and 8 × 10^11^ to achieve sparse infection.

### Stereotactic injections

#### Juvenile injection

P1 to P3 pups were anaesthetized with isoflurane for juvenile injections and mounted on a stereotactic frame (Stoelting). Mice used for vTRAP or RNA-seq experiments received six 150 nl injections at a speed of 10 nl s^−1^ in S1 in the left hemisphere. All other mice received three 150 nl injections at a speed of 10 nl s^−1^ in S1 in the left hemisphere. For vTRAP experiments, viruses were supplemented with green fluorescent beads (LumaFluor) to label the injection sites. Mice were allowed to recover in a heated recovery chamber (Vettech, HEO11) before returning to the home cage.

#### Adult injections

P42 to P60 mice were anaesthetized with isoflurane and mounted on a stereotactic frame. A small incision was made in the skin over the injection area on the right hemisphere, and the skull was cleaned using a cotton swab. Two holes were drilled using a rotary drill (Foredom, K.1070) at the following coordinates (from Bregma): (1) anteroposterior +1.0 mm and mediolateral −3.2 mm; and (2) anteroposterior +1.5 mm and mediolateral −3.2 mm for injections in S1, or (1) anteroposterior -2.0 mm and mediolateral −1.7 mm; and (2) anteroposterior −1.5 mm and mediolateral −1.4 mm for injection in hippocampal CA1. Mice received two 300 nl injections at a 100 nl min^−1^ speed at each location (depth S1: 0.3 mm, depth CA1: 1.2 mm). After injection, the injection capillary was left in place for three minutes before retraction. Following injections, the skin was sutured with absorbable Vicryl sutures (Ethicon, W9500T), and mice were allowed to recover in a heated recovery chamber at 36 °C before being returned to the home cage.

### Electrophysiology

#### Slice preparation

Mice (P42 to P70) were deeply anesthetised with an overdose of sodium pentobarbital and transcardially perfused with 10 ml ice-cold *N*-methyl-d-glucamine (NMDG) solution containing (in mM) 93 NMDG, 2.5 KCl, 1.2 NaH_2_PO_4_, 30 NaHCO_3_, 20 HEPES, 25 glucose, 5 sodium ascorbate, 2 thiourea, 3 sodium pyruvate, 10 MgSO_4_ and 0.5 CaCl_2_ (300–310 mOsm, pH 7.3–7.4) oxygenated with 95% O_2_ and 5% CO_2_. Following decapitation, the brain was quickly removed, and the injected hemisphere was glued to a cutting platform before submerging in ice-cold NMDG solution. Coronal slices (300 µm) were cut using a vibratome (VT1200S, Leica) and placed in NMDG solution at 32 °C for 11 min before being transferred to a holding solution containing (in mM) 92 NaCl, 2.5 KCl, 1.2 NaH_2_PO_4_, 30 NaHCO_3_, 20 HEPES, 25 glucose, 5 sodium ascorbate, 2 thiourea, 3 sodium pyruvate, 2 MgSO_4_ and 2 CaCl_2_ (300–310 mOsm, pH 7.3–7.4), oxygenated with 95% O_2_ and 5% CO_2_, at room temperature for at least 45 min until recording. All salts were purchased from Sigma-Aldrich.

#### Electrophysiological recordings

Slices were transferred to the recording setup 15 min prior to the recording while being continuously superfused with recording artificial cerebrospinal fluid (ACSF) containing (in mM) 124 NaCl, 1.25 NaH_2_PO_4_, 3 KCl, 26 NaHCO_3_, 10 Glucose, 2 CaCl_2_, and 1 MgCl_2_, continuously oxygenated with 95% O_2_ and 5% CO_2_ at 32 °C. Pipettes (3–5 MΩ) were made from borosilicate glass capillaries using a PC-10 pipette puller (P10, Narishige) and filled with intracellular solution containing (in mM) 115 CsMeSO_3_, 20 CsCl, 10 HEPES, 2.5 MgCl_2_, 4 Na_2_ATP, 0.4 Na_3_GTP, 10 sodium-phosphocreatine, 0.6 EGTA (pH 7.2–7.3, 285–295 mOsm). Cells were visualized with an upright microscope (Olympus) and recorded using a Multiclamp 700B amplifier (Molecular Devices). The signal was passed through a Hum Bug Noise Eliminator (Digitimer), sampled at 20 kHz, filtered at 3 kHz using a Digidata 1440 A (Molecular Devices) and recorded using Clampex 10.7 (Molecular Devices). All cells were recorded in S1 layer 2/3. mEPSCs and mIPSCs were recorded in the presence of 1 µM tetrodotoxin (Tocris, 1069) at a holding voltage of −60 mV and +10 mV, respectively. Synaptic currents were not blocked to record mEPSCs and mIPSCs from the same cells. Cells were excluded if the access resistance (*R*_a_) exceeded 25 MΩ or holding current (*I*_hold_) > 200 pA (at holding voltage (*V*_hold_) = −60 mV). For ChR2 eIPSCs, slices were incubated in recording ACSF supplemented with 5 μM 6-cyano-7-nitroquinoxaline-2,3-dione (CNQX, Tocris, 1045) and 100 μM d-(−)-2-amino-5-phosphonopentanoic acid (d-APV, Tocris, 0106) to block glutamatergic inputs. Cells were held at a holding voltage of +10 mV, and after allowing the cell to settle (approximately 1 min), sIPSCs were recorded. Subsequently, whole-cell series resistance was compensated by 70%. eIPSC were evoked with full-field 5 ms LED pulses (power: PV–PV 10% LED, SOM–PV 10% LED, VIP–PV 100% LED, as indicated for the respective experiments. 100% LED power = 75.1 mW mm^−2^) through a 4× objective (Olympus) with an inter-stimulus interval of 60 s from a pE100 illumination system (CoolLED). Cells were excluded from analysis if the series resistance changed >20%, *R*_a_ >25 MΩ or *I*_hold_ > 200 pA (at *V*_hold_ = −60 mV), and only cells with both sIPSC and eIPSC recordings were included in the data. Cell-attached recordings were performed using recording electrodes filled with recording ACSF in the presence of CNQX and d-APV, loose seals (around 200 MΩ) were formed on cells, and LED stimulus of various intensities was applied via a 4× objective in the same way as in the eIPSC recordings (see above). Paired-pulse ratios were recorded following stimulation in S1 layer 2/3 using an ISO-STIM 01D stimulator (NPI) and a tungsten stimulation electrode (TST33A20KT, WPI) in the presence of CNQX and d-APV at a holding potential of +10 mV. Stimulation strength was set to evoke a ~300 pA response to the first stimulus. Two 1 ms pulses were given with a 50 ms inter-stimulus interval. PPR was calculated as peak 2/peak 1 after correcting for any residual current at the second pulse.

#### Electrophysiology analysis

Miniature (mEPSC and mIPSC) and spontaneous (sIPSC) recordings were analysed using Mini Analysis (version 6.0.7, Synaptosoft). *E*/*I* ratio was calculated as (excitatory current/second)/(inhibitory current/second). Current per second was calculated as (average charge of an event) × frequency. *E*/*I* ratios were calculated for each recorded cell, and only cells with mEPSC and mIPSC recordings that passed quality control (see above) were included. eIPSC, cell-attached and paired-pulse ratio recordings were analysed in Clampfit 10.2 (Molecular Devices).

### Biochemistry and RNA-seq

#### RNA isolation by anti-HA pulldown

The infected cortex from P44 to P70 mice, injected with AAV8-hSyn-flx-fDIO-HA-Rpl10a-T2A-Myc-hM3D(Gq) and treated for 48 h with vehicle or CNO, was rapidly dissected in ice-cold RNase-free PBS, using fluorescent beads as a guide to identifying the infected area, and immediately homogenized in ice-cold homogenization buffer (50 mM Tris-HCl pH 7.5, 100 mM KCl, 12 mM MgCl2, 1 mg ml^−1^ Heparin (Sigma-Aldrich), cOmplete EDTA-free protease inhibitors (Sigma-Aldrich), 200 U ml^−1^ RNAsin (Promega), 100 µg ml^−1^ cycloheximide and 1 mM DTT (Sigma-Aldrich)). Tissue from 4 brains was pooled for every sample. Samples were centrifuged at 2,000*g* for 10 min at 4 °C, and Igepal-CA630 (Sigma-Aldrich) was added to the samples to a final concentration of 1%. Samples were then centrifuged at 13,000*g* for 10 min at 4 °C, and the supernatant was added on 100 µl of anti-HA magnetic beads (Pierce 88837, previously washed in homogenization buffer) for 3–4 h at 4 °C with gentle rotation. After incubation, beads were washed 3 times in ice-cold washing buffer (300 mM KCl, 1% Igepal-CA630, 50 mM Tris-HCl pH 7.5, 12 mM MgCl2, 1 mM DTT and 100 µg ml^−1^ cycloheximide) and eluted in 350 µl of RLT Plus buffer from the RNAeasy Plus Micro kit (Qiagen) supplemented with 2-mercaptoethanol (Bio-Rad).

#### RNA purification, quantification, and quality check

RNA purification of immunoprecipitated RNA was performed using the RNeasy Plus Micro kit (Qiagen) following the manufacturer’s protocol. RNA quality was checked on a Bioanalyzer instrument (Agilent Technologies) using an RNA 6000 Pico Chip. Only RNA samples with RNA integrity number (RIN) values higher than 9 were used for library preparation and sequencing.

#### Library preparation and Illumina sequencing

Four biological replicates were analysed for each genotype. The Genomic Unit of the Centre for Genomic Regulation (CRG, Barcelona, Spain) performed the library preparation and RNA-seq. The library was prepared using the SMARTer Ultra Low RNAkit and samples were then sequenced paired-end using an Illumina HiSeq 2500 platform to a mean of approximately 60 million mapped reads per sample.

#### Bioinformatics

High-throughput sequencing data from PV^+^ interneurons-derived ribosome-associated mRNAs were processed using the community-curated Nextflow (version 21.03.0.edge, build 5518 (3 May 2021 10:52 UTC), available at https://zenodo.org/record/3490660#.Y8AhHXbP2Uk) RNA-seq pipeline^76^. Specifically, sequencing reads were quality-controlled by FastQC (available at https://www.bioinformatics.babraham.ac.uk/projects/fastqc/) and quality-trimmed by Trim Galore (available at https://zenodo.org/record/5127899#.Y8fdOi-l3UI). Mouse GRCm38/mm10 genome annotation was accessed from Illumina’s iGenomes repository (available at https://support.illumina.com/sequencing/sequencing_software/igenome.html) and used as a reference for read alignment by STAR^77^ and for gene abundance quantification by Salmon^78^. Gene-level counts data were imported into R using the tximport package^79^ and analysed by edgeR^80^ using the estimateGLMRobustDisp model. Genes with less than 10 reads in at least 4 samples were excluded from the analysis^81^. The >1.5-fold, false discovery rate < 0.05 and transcript per million >1 cut-off were used to identify genes that respond to PV^+^ interneuron activation (Extended Data Table 1). Activity-dependent genes were ranked using four scoring criteria: fold change, CoV, STRING (https://string-db.org/) and secretome scores. The fold-change score indicates the magnitude of the response and is scaled for a value between 0 and 1. The CoV score represents the degree of reproducibility and was calculated by scaling the sum of the ratio of the standard deviation to the mean from each sample. The STRING score of each gene signifies its co-interaction with other activation-response genes and was computed by scaling the sum of interaction scores obtained from STRING enrichment analysis^42^. The secretome score takes up a binary value. Genes that are part of the human secretome^82^ were assigned a value of 1.

### Statistical analyses

All statistical analyses were performed using SPSS (Supplementary table 1). No statistical methods were used to predetermine sample sizes. Sample sizes were chosen on the basis of previous publications in the field. Experimental mice from all genotypes or conditions were processed together. Samples were tested for normality using the Shapiro–Wilk normality test. Differences were considered significant when *P* < 0.05. Data are presented as mean ± s.e.m. Statistical details of experiments are described in figure legends.

### Reporting summary

Further information on research design is available in the Nature Portfolio Reporting Summary linked to this article.

## Online content

Any methods, additional references, Nature Portfolio reporting summaries, source data, extended data, supplementary information, acknowledgements, peer review information; details of author contributions and competing interests; and statements of data and code availability are available at 10.1038/s41586-025-08933-z.

## Supplementary information

Supplementary Table 1 Summary of data and statistical analyses

Reporting Summary

## Source data

Source Data Fig. 1

Source Data Fig. 2

Source Data Fig. 3

Source Data Fig. 4

Source Data Fig. 5

Source Data Extended Data Fig. 1

Source Data Extended Data Fig. 2

Source Data Extended Data Fig. 3

Source Data Extended Data Fig. 4

Source Data Extended Data Fig. 5

Source Data Extended Data Fig. 6

Source Data Extended Data Fig. 7

Source Data Extended Data Fig. 8

Source Data Extended Data Fig. 9

Source Data Extended Data Fig. 10

## Figures and Tables

**Extended Data Table 1:**
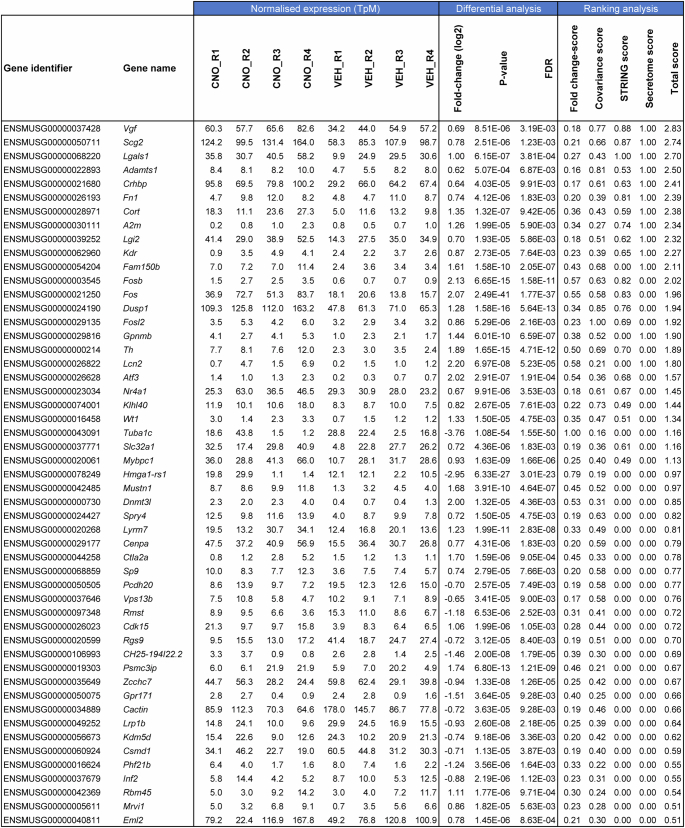
Genes up- and downregulated following PV+ interneuron activation

FDR: False discovery rate. P-values were calculated using a two-sided Wald’s test with adjustment for multiple testing.

## Data Availability

RNA-seq data have been deposited to the Gene Expression Omnibus (GEO) under accession GSE223038. Other data are available on King’s College London Open Research Data System at 10.18742/28334123. Source data are provided with this paper.

## Code availability

The source code for the processing of RNA-seq data are available on GitHub at https://github.com/fursham-h/Nature_Selten_Supplementary.
