# Supplementary material for: Regulation of PV interneuron plasticity by neuropeptide-encoding genes
Source: Nature. 2025 Apr 30;643(8070):173–81. doi: 10.1038/s41586-025-08933-z (PMC12222018; doi:10.1038/s41586-025-08933-z)
Supplement: Supplementary file 1 — Summary of data and statistical analyses [file 41586_2025_8933_MOESM1_ESM.pdf]

nature portfolio

https://doi.org/10.1038/s41586-025-08933-z

Supplementary information

Regulation of PV interneuron plasticity by neuropeptide-encoding genes

In the format provided by the
authors and unedited

Supplementary Table 1. Summary of data and statistical analyses.

Figure 1	Measurement (unit)	Values	N [unit]	Statistical test	P-value
Figure 1c	Fos intensity (a.u.)	Vehicle: 108.3 ± 9.4; CNO: 147.8 ± 5.15	[brains] Vehicle, n = 3; CNO, n = 3	Two-tailed Student's t-test	0.02
Figure 1e	mEPSC amplitude (pA) and frequency (Hz)	Amplitude Vehicle: 14.3 ± 0.6; CNO: 14.6 ± 0.3<br><br>Frequency Vehicle 10.6 ± 1.0; CNO: 11.7 ± 1.1	[cells, slices, brains] Vehicle, n = 13, 10, 5; CNO, n = 18, 15, 8	Two-tailed Student's t-test	Amplitude: 0.71<br><br>Frequency: 0.48
Figure 1g	mIPSC amplitude (pA) and frequency (Hz)	Amplitude Vehicle: 15.2 ± 0.7; CNO: 19.0 ± 0.9<br><br>Frequency Vehicle 7.6 ± 1.1; CNO: 14.1 ± 1.4	[cells, slices, brains] Vehicle, n = 13, 10, 5; CNO, n = 18, 15, 8	Amplitude: Two-tailed Student's t-test<br><br>Frequency: Mann-Whitney U-test	Amplitude: 0.003<br><br>Frequency: < 0.001
Figure 1h	E/I ratio	Vehicle: 0.25 ± 0.03; CNO: 0.15 ± 0.02	[cells, slices, brains] Vehicle, n = 13, 10, 5; CNO, n = 18, 15, 8	Two-tailed Student's t-test	0.005
Figure 1j	Synaptic density change (%)	Vehicle: 5.1 ± 6.6; CNO: 39.0 ± 8.7	[brains] Vehicle, n = 8; CNO, n = 9	Two-tailed Student's t-test	0.008
**Figure 2**	**Measurement (unit)**	**Values**	**N [unit]**	**Statistical test**	**P-value**
Figure 2d	Peak amplitude (nA) and charge (pC)	Peak amplitude Vehicle: 0.7 ± 0.1; CNO: 1.1 ± 0.1<br><br>Charge Vehicle: 10.89 ± 1.90; CNO: 16.69 ± 1.74	[cells, slices, brains] Vehicle, n = 13, 13, 7; CNO, n = 11, 11, 7	Two-tailed Student's t-test	Peak amplitude: 0.01<br><br>Charge: 0.04
Figure 2g	Peak amplitude (nA) and charge (pC)	Peak amplitude Vehicle: 0.3 ± 0.03; CNO: 0.4 ± 0.07<br><br>Charge Vehicle: 11.91 ± 1.47; CNO: 14.23 ± 2.79	[cells, slices, brains] Vehicle, n = 10, 10, 6; CNO, n = 11, 11, 5	Two-tailed Student's t-test	Peak amplitude: 0.64<br><br>Charge: 0.48
Figure 2j	Peak amplitude (nA) and charge (pC)	Peak amplitude Vehicle: 0.078 ± 0.02; CNO: 0.067 ± 0.01<br><br>Charge Vehicle: 2.81 ± 0.88; CNO: 1.70 ± 0.23	[cells, slices, brains] Vehicle, n = 13, 12, 6<br><br>CNO, n = 12, 10, 5	Two-tailed Student's t-test	Peak amplitude: 0.67<br><br>Charge: 0.25
**Figure 3**	**Measurement (unit)**	**Values**	**N [unit]**	**Statistical test**	**P-value**
Figure 3f	*Scg2* mRNA/cell (a.u.) and *Vgf* mRNA/cell (a.u.)	*Scg2* mRNA/cell Uninfected: 42.0 ± 3.8; Infected: 71.5 ± 6.6<br><br>*Vgf* mRNA/cell Uninfected: 39.5 ± 2.1; Infected: 73.5 ± 9.8	[brains] *Scg2*: n = 6; *Vgf*: n=5	Two-tailed paired Student's t-test	Scg2: 0.004<br><br>Vgf: 0.01
**Figure 4**	**Measurement (unit)**	**Values**	**N [unit]**	**Statistical test**	**P-value**
Figure 4c	Synaptic density change (%)	shLacZ, 24.5 ± 6.4; shScg2, -12.4 ± 5.2; shVgf, -1.8 ± 5.9	[brains] shLacZ, n = 10; shScg2, n = 9; shVgf, n = 8	Two-tailed One-sample t-test	*shScg2*: 0.049<br><br>*ShVgf*: 0.007
Figure 4f	Synaptic density change (%)	mCherry: -10.0 ± 6.41; Vgf-mCherry: 36.9 ± 8.80	[brains]	Two-tailed Student's t-test	0.003

			mCherry, n = 5; Vgf-mCherry, n = 5		
**Figure 5**	**Measurement (unit)**	**Values**	**N [unit]**	**Statistical test**	**P-value**
Figure 5c	Synaptic density (puncta/µm)	Fos$^-$, 0.129 ± 0.012; Fos$^+$, 0.101 ± 0.003	[brains]<br>n = 5	Two-tailed paired Student's t-test	0.044
Figure 5f	*Vgf* intensity (a.u.)	tdTomato$^-$: 43.1 ± 6.4; tdTomato$^+$: 92.4 ± 9.9	[brains]<br>n = 5	Two-tailed paired Student's t-test	< 0.001
Figure 5h	Synaptic density (puncta/µm)	24h:<br>tdTomato$^-$: 0.146 ± 0.010; tdTomato$^+$: 0.096 ± 0.011<br><br>72h: tdTomato$^-$: 0.153 ± 0.009; tdTomato$^+$: 0.143 ± 0.015	[brains]<br>24h, n = 5<br>72h, n = 5	Two-tailed paired Student's t-test<br><br>Two-tailed Student's t-test	24h: 0.001; 72h: 0.59<br><br>tdTomato$^-$0.65; tdTomato$^+$ 0.033
**Extended Data Figure 1**	**Measurement (unit)**	**Values**	**N [unit]**	**Statistical test**	**P-value**
Extended Data Figure 1b	Infection density (%)	28.1 ± 3.1	[brains]<br>n = 6	n.a.	n.a.
Extended Data Figure 1e	mEPSC amplitude (pA) and frequency (Hz)	Amplitude<br>Vehicle: 13.3 ± 0.5; CNO: 13.7 ± 0.5<br><br>Frequency<br>Vehicle: 12.7 ± 1.7; CNO: 11.1 ± 0.9	[cells, slices, brains]<br>Vehicle, n = 9, 9, 3; CNO, n = 10, 6, 3	Two-tailed Student's t-test	Amplitude: 0.51<br><br>Frequency: 0.45
Extended Data Figure 1g	mIPSC amplitude (pA) and frequency (Hz)	Amplitude<br>Vehicle: 15.5 ± 0.62; CNO: 15.0 ± 0.77<br><br>Frequency<br>Vehicle: 10.6 ± 1.8; CNO: 11.1 ± 1.3	[cells, slices, brains]<br>Vehicle, n = 9, 9, 3; CNO, n = 10, 6, 3	Two-tailed Student's t-test	Amplitude: 0.64<br><br>Frequency: 0.84
Extended Data Figure 1h	E/I ratio	Vehicle: 0.27 ± 0.04; CNO: 0.22 ± 0.02	[cells, slices, brains]<br>Vehicle, n = 9, 9, 3; CNO, n = 10, 6, 3	Two-tailed Student's t-test	0.24
**Extended Data Figure 2**	**Measurement (unit)**	**Values**	**N [unit]**	**Statistical test**	**P-value**
Extended Data Figure 2b	Synaptic density (puncta/µm)	Uninfected: 0.106 ± 0.016; Infected: 0.104 ± 0.010	[brains]<br>n = 8	Two-tailed paired Student's t-test	0.86
Extended Data Figure 2d	Synaptic density (puncta/µm)	Uninfected: 0.091 ± 0.010; Infected: 0.121 ± 0.010	[brains]<br>n = 9	Two-tailed paired Student's t-test	$8x10^{-4}$
Extended Data Figure 2e	Synaptic density (puncta/µm)	Vehicle: 0.106 ± 0.016; CNO: 0.091 ± 0.010	[brains]<br>Vehicle, n = 8; CNO, n = 9	Two-tailed Student's t-test	0.44
Extended Data Figure 2h	mEPSC amplitude (pA) and frequency (Hz)	Amplitude<br>Vehicle: 14.9 ± 0.5; CNO: 16.0 ± 0.7<br><br>Frequency<br>Vehicle: 10.3 ± 0.7; CNO: 9.5 ± 0.7	[cells, slices, brains]<br>Vehicle, n = 15, 14, 7; CNO, n = 16, 12, 7	Two-tailed Student's t-test	Amplitude: 0.20<br><br>Frequency: 0.46
Extended Data Figure 2j	mIPSC amplitude (pA) and frequency (Hz)	Amplitude<br>Vehicle: 17.3 ± 0.75; CNO: 15.2 ± 1.0<br><br>Frequency	[cells, slices, brains]<br>Vehicle, n = 15, 14, 7; CNO, n = 16, 12, 7	Two-tailed Student's t-test	Amplitude: 0.14<br><br>Frequency: 0.03

		Vehicle: 10.1 ± 1.0; CNO: 7.2 ± 0.7			
Extended Data Figure 2k	E/I ratio	Vehicle: 0.19 ± 0.02; CNO: 0.30 ± 0.03	[cells, slices, brains] Vehicle, n = 15, 14, 7; CNO, n = 16, 12, 7	Two-tailed Student's t-test	0.009
**Extended Data Figure 3**	**Measurement (unit)**	**Values**	**N [unit]**	**Statistical test**	**P-value**
Extended Data Figure 3b	Infection density (%)	16.4 ± 2.7	[brains] n = 8	n.a.	n.a.
Extended Data Figure 3d	Fos intensity (a.u.)	Vehicle: 181.5 ± 11.3; CNO: 233.1 ± 15.1	[brains] Vehicle, n = 4; CNO, n = 4	Two-tailed Student's t-test	0.03
Extended Data Figure 3f	Charge (nC)	-60 mV: 12.2 ± 2.1; +10 mV: 2.0 ± 0.5	[cells, slices, brains] n = 4, 3, 2	Two-tailed paired Student's t-test	0.01
Extended Data Figure 3j	Charge (nC)	Vehicle: 75.2 ± 26.3; PTX: -2.0 ± 0.5	[cells, slices, brains] n = 4, 4, 2	Two-tailed Student's t-test	0.03
**Extended Data Figure 4**	**Measurement (unit)**	**Values**	**N [unit]**	**Statistical test**	**P-value**
Extended Data Figure 4b	Number of action potentials (n)	Vehicle: 5% LED, 0.9 ± 0.2; 10% LED, 1.4 ± 0.3; 25% LED, 2.3 ± 0.4; 50% LED, 2.9 ± 0.4; 100% LED, 3.7 ± 0.4<br><br>CNO: 5% LED, 0.9 ± 0.2; 10% LED, 1.6 ± 0.2; 25% LED, 2.1 ± 0.3; 50% LED, 2.4 ± 0.3; 100% LED, 3.1 ± 0.4	[cells, slices, brains] Vehicle, n = 10, 9, 5; CNO, n = 11, 10, 7	Two-tailed Student's t-test	5% LED: 0.97<br><br>10% LED: 0.59<br><br>25% LED: 0.76<br><br>50% LED: 0.32<br><br>100% LED: 0.29
Extended Data Figure 4c	Number of action potentials (n)	Vehicle: 1.4 ± 0.3; CNO: 1.6 ± 0.2	[cells, slices, brains] Vehicle, n = 10, 9, 5; CNO, n = 11, 10, 7	Two-tailed Student's t-test	0.59
Extended Data Figure 4e	Number of action potentials (n)	Vehicle: 5% LED, 0.9 ± 0.2; 10% LED, 1.1 ± 0.3; 25% LED, 1.9 ± 0.3; 50% LED, 2.5 ± 0.4; 100% LED, 3.0 ± 0.5<br><br>CNO: 5% LED, 0.5 ± 0.2; 10% LED, 1.1 ± 0.2; 25% LED, 1.4 ± 0.3; 50% LED, 1.4 ± 0.2; 100% LED, 1.7 ± 0.3	[cells, slices, brains] Vehicle, n = 10, 9, 5; CNO, n = 10, 5, 6	Two-tailed Student's t-test	5% LED: 0.23<br><br>10% LED: 1.00<br><br>25% LED: 0.21<br><br>50% LED: 0.04<br><br>100% LED: 0.04
Extended Data Figure 4f	Number of action potentials (n)	Vehicle: 11.1 ± 0.3; CNO: 1.1 ± 0.2	[cells, slices, brains] Vehicle, n = 10, 9, 5; CNO, n = 10, 5, 6	Two-tailed Student's t-test	1.00
Extended Data Figure 4h	Number of action potentials (n)	Vehicle: 2% LED, 0.7 ± 0.1; 3% LED, 0.8 ± 0.1; 5% LED, 1.0 ± 0.1; 10% LED, 1.6 ± 0.2; 25% LED, 2.4 ± 0.2; 50% LED, 2.9 ± 0.3; 100% LED, 3.5 ± 0.3	[cells, slices, brains] Vehicle, n = 13, 8, 5; CNO, n = 8, 5, 2	Two-tailed Student's t-test	2% LED:0.65<br><br>3% LED: 0.61<br><br>5% LED: 0.59<br><br>10% LED: 0.67

		CNO:<br>2% LED, 0.8 ± 0.2; 3% LED, 0.8 ± 0.2; 5% LED, 1.1 ± 0.2; 10% LED, 1.8 ± 0.3; 25% LED, 2.1 ± 0.4; 50% LED, 2.6 ± 0.3; 100% LED, 3.1 ± 0.4			25% LED: 0.48<br><br>50% LED: 0.55<br><br>100% LED: 0.42
Extended Data Figure 4i	Number of action potentials (n)	Vehicle: 1.6 ± 0.2; CNO: 1.8 ± 0.3	[cells, slices, brains]<br>Vehicle, n = 13, 8, 5; CNO, n = 8, 5, 2	Two-tailed Student's t-test	0.67
Extended Data Figure 4k	Number of action potentials (n)	Vehicle:<br>2% LED, 0.5 ± 0.2; 3% LED, 1.6 ± 0.5; 5% LED, 2.6 ± 0.5; 10% LED, 3.2 ± 0.4; 25% LED, 4.9 ± 0.6; 50% LED, 5.7 ± 0.7; 100% LED, 6.6 ± 0.8<br><br>CNO:<br>2% LED, 0.7 ± 0.2; 3% LED, 1.5 ± 0.3; 5% LED, 2.6 ± 0.4; 10% LED, 3.7 ± 0.4; 25% LED, 4.9 ± 0.5; 50% LED, 5.9 ± 0.6; 100% LED, 6.9 ± 0.7	[cells, slices, brains]<br>Vehicle, n = 15, 10, 3; CNO, n = 17, 10, 3	Two-tailed Student's t-test	2% LED: 0.55<br><br>3% LED: 0.77<br><br>5% LED: 0.94<br><br>10% LED: 0.38<br><br>25% LED: 0.98<br><br>50% LED: 0.85<br><br>100% LED: 0.69
Extended Data Figure 4l	Number of action potentials (n)	Vehicle: 6.6 ± 0.8; CNO: 6.9 ± 0.7	[cells, slices, brains]<br>Vehicle, n = 15, 10, 3; CNO, n = 17, 10, 3	Two-tailed Student's t-test	0.69
Extended Data Figure 4o	Peak amplitude (nA) and charge (pC)	Peak amplitude<br>Vehicle: 4.3 ± 0.6; CNO: 3.9 ± 0.8<br><br>Charge<br>Vehicle: 84.0 ± 10.2; CNO: 75.7 ± 13.5	[cells, slices, brains]<br>Vehicle, n = 13, 13, 5; CNO, n = 12, 12, 5	Two-tailed Student's t-test	Peak amplitude: 0.76<br><br>Charge: 0.66
**Extended Data Figure 5**	**Measurement (unit)**	**Values**	**N [unit]**	**Statistical test**	**P-value**
Extended Data Figure 5b	sIPSC amplitude (pA) and frequency (Hz)	Amplitude<br>Vehicle: 22.0 ± 1.5; CNO: 30.6 ± 2.2<br><br>Frequency<br>Vehicle 10.5 ± 1.6; CNO: 24.4 ± 2.8	[cells, slices, brains]<br>Vehicle, n = 13, 13, 7; CNO, n = 11, 11, 7	Two-tailed Student's t-test	Amplitude: 0.003<br><br>Frequency: 1.1×10^{-7}
Extended Data Figure 5d	sIPSC amplitude (pA) and frequency (Hz)	Amplitude<br>Vehicle: 56.4 ± 6.4; CNO: 47.4 ± 4.6<br><br>Frequency<br>Vehicle 25.0 ± 1.9; CNO: 27.8 ± 2.9	[cells, slices, brains]<br>Vehicle, n = 13, 13, 5; CNO, n = 12, 12, 5	Two-tailed Student's t-test	Amplitude: 0.28<br><br>Frequency: 0.43
Extended Data Figure 5f	sIPSC amplitude (pA) and frequency (Hz)	Amplitude<br>Vehicle: 27.2 ± 1.8; CNO: 43.3 ± 5.0<br><br>Frequency<br>Vehicle 15.0 ± 1.7; CNO: 24.5 ± 2.4	[cells, slices, brains]<br>Vehicle, n = 10, 10, 6; CNO, n = 11, 11, 5	Two-tailed Student's t-test	Amplitude: 0.009<br><br>Frequency: 0.004
Extended Data Figure 5h	sIPSC amplitude (pA) and frequency (Hz)	Amplitude<br>Vehicle: 23.7 ± 1.8; CNO: 29.7 ± 1.8	[cells, slices, brains]	Two-tailed Student's t-test	Amplitude: 0.03<br><br>Frequency:

		Frequency Vehicle 12.7 ± 2.0; CNO: 22.6 ± 2.7	Vehicle, n = 13, 12, 6; CNO, n = 12, 10, 5		0.006
**Extended Data Figure 6**	**Measurement (unit)**	**Values**	**N [unit]**	**Statistical test**	**P-value**
Extended Data Figure 6b	Synaptic density change (%)	Vehicle: 0.981 ± 0.108; CNO: 1.423 ± 0.095	[brains] Vehicle, n = 6; CNO, n = 6	Two-tailed Student's t-test	0.01
Extended Data Figure 6d	Synaptic density (puncta/µm)	Uninfected: 0.100 ± 0.005; Infected: 0.100 ± 0.010	[brains] n = 6	Two-tailed paired Student's t-test	0.79
Extended Data Figure 6f	Synaptic density (puncta/µm)	Uninfected: 0.098 ± 0.006; Infected: 0.138 ± 0.007	[brains] n = 6	Two-tailed paired Student's t-test	0.006
Extended Data Figure 6g	Synaptic density (puncta/µm)	Vehicle: 0.100 ± 0.005 CNO: 0.098 ± 0.006	[brains] Vehicle, n = 6; CNO, n = 6	Two-tailed Student's t-test	0.87
Extended Data Figure 6i	Infection density (%)	22.3 ± 3.8	[brains] n = 7	n.a.	n.a.
Extended Data Figure 6k	Fos intensity (a.u.)	Vehicle: 139.7 ± 7.9; CNO: 328.5 ± 42.9	[brains] Vehicle, n = 4; CNO, n = 4	Two-tailed Student's t-test	0.004
**Extended Data Figure 7**	**Measurement (unit)**	**Values**	**N [unit]**	**Statistical test**	**P-value**
Extended Data Figure 7c	*Scg2* mRNA/cell (a.u.)	Uninfected: 38.6 ± 5.2; Infected: 37.7 ± 4.9	[brains] Scg2: n = 6	Two-tailed paired Student's t-test	0.71
Extended Data Figure 7e	*Vgf* mRNA/cell (a.u.)	Uninfected: 37.3 ± 3.8; Infected: 40.0 ± 1.8	[brains] Vgf: n=6	Two-tailed paired Student's t-test	0.58
Extended Data Figure 7h	*Scg2* mRNA/cell (a.u.) and *Vgf* mRNA/cell (a.u.)	*Scg2* mRNA/cell Uninfected: 78.8 ± 5.4; Infected: 59.1 ± 5.8<br><br>*Vgf* mRNA/cell Uninfected: 43.3 ± 3.8; Infected: 33.5 ± 4.1	[brains] *Scg2*: n = 5<br><br>*Vgf*: n=5	Two-tailed paired Student's t-test	*Scg2*: 0.007<br><br>*Vgf*: 0.02
Extended Data Figure 7j	Infection density (%)	*shLacZ-hM3Dq*: 15.5 ± 2.6<br><br>*shScg2-hM3Dq*: 36.6 ± 8.1<br><br>*shVgf-hM3Dq*: 13.1 ± 1.9	[brains] shLacZ-hM3Dq n = 3<br><br>shLacZ-hM3Dq n = 5<br><br>shLacZ-hM3Dq n = 5	n.a.	n.a.
Extended Data Figure 7k	Fos intensity (a.u.)	shLacZ-hM3Dq: Vehicle, 28.3 ± 4.8 CNO, 59.1 ± 6.1<br><br>shScg2-hM3Dq: Vehicle, 28.4 ± 1.0 CNO, 50.4 ± 2.5<br><br>shVgf-hM3Dq: Vehicle, 9.7 ± 5.1 CNO, 28.6 ± 3.8	[brains] shLacZ-hM3Dq Vehicle, n = 3 CNO, n = 3<br><br>shLacZ-hM3Dq Vehicle, n = 3 CNO, n = 3<br><br>shLacZ-hM3Dq Vehicle, n = 3 CNO, n = 3	Two-tailed Student's t-test	shLacZ-hM3Dq: 0.02<br><br>shScg2-hM3Dq: 0.001<br><br>shVgf-hM3Dq: 0.04
**Extended Data Figure 8**	**Measurement (unit)**	**Values**	**N [unit]**	**Statistical test**	**P-value**
Extended Data Figure 8b	Synaptic density change (%)	shLacZ, 0.0 ± 9.1<br><br>shScg2, 8.7 ± 7.4	[brains]	Two-tailed One-sample t-test	shScg2: 0.27

		shVgf, -0.8 ± 9.8	shLacZ, n = 8; shScg2, n = 9; shVgf, n = 7		shVgf: 0.93
Extended Data Figure 8d	Synaptic density (puncta/µm)	shLacZ-hM3Dq:<br>Vehicle:<br>Uninfected, 0.13 ± 0.01<br>Infected, 0.13 ± 0.01<br><br>CNO:<br>Uninfected, 0.11 ± 0.01<br>Infected, 0.14 ± 0.01	[brains] shLacZ-hM3Dq<br>Vehicle, n = 8<br>CNO, n = 10	Two-tailed paired Student's t-test	shLacZ-hM3Dq:<br>Vehicle:<br>0.67<br><br>CNO<br>0.003
Extended Data Figure 8f	Synaptic density (puncta/µm)	shScg2-hM3Dq:<br>Vehicle:<br>Uninfected, 0.12 ± 0.01<br>Infected, 0.13 ± 0.01<br><br>CNO:<br>Uninfected, 0.13 ± 0.01<br>Infected, 0.14 ± 0.01	[brains] shLacZ-hM3Dq<br>Vehicle, n = 9;<br>CNO, n = 9	Two-tailed paired Student's t-test	shScg2-hM3Dq:<br>Vehicle:<br>0.40<br><br>CNO:<br>0.07
Extended Data Figure 8h	Synaptic density (puncta/µm)	shVgf-hM3Dq:<br>Vehicle:<br>Uninfected, 0.13 ± 0.01<br>Infected, 0.12 ± 0.01<br><br>CNO:<br>Uninfected, 0.13 ± 0.01<br>Infected, 0.13 ± 0.01	[brains] shLacZ-hM3Dq<br>Vehicle, n = 7;<br>CNO, n = 8	Two-tailed paired Student's t-test	shVgf-hM3Dq:<br>Vehicle:<br>0.70<br><br>CNO:<br>0.72
**Extended Data Figure 9**	**Measurement (unit)**	**Values**	**N [unit]**	**Statistical test**	**P-value**
Extended Data Figure 9c	mRNA/cell (a.u.)	Uninfected, 25.9 ± 3.8<br>Infected, 109.1 ± 14.4	[brains]<br>n = 3	Two-tailed paired Student's t-test	0.03
Extended Data Figure 9e	Synaptic density (puncta/µm)	Uninfected, 0.11 ± 0.10<br>Infected, 0.10 ± 0.01	[brains]<br>n = 5	Two-tailed paired Student's t-test	0.23
Extended Data Figure 9g	Synaptic density (puncta/µm)	Uninfected, 0.09 ± 0.01<br>Infected, 0.12 ± 0.01	[brains]<br>n = 5	Two-tailed paired Student's t-test	0.01
Extended Data Figure 9i	mEPSC amplitude (pA) and frequency (Hz)	Amplitude<br>mCherry: 15.5 ± 0.8;<br>VGF-mCherry: 15.0 ± 0.5<br><br>Frequency<br>mCherry: 15.5 ± 1.8;<br>VGF-mCherry: 20.1 ± 0.5	[cells, slices, brains]<br>mCherry,<br>n = 9, 9, 4; VGF-mCherry,<br>n = 7, 7, 4	Two-tailed Student's t-test	Amplitude:<br>0.59<br><br>Frequency:<br>0.12
Extended Data Figure 9k	mIPSC amplitude (pA) and frequency (Hz)	Amplitude<br>mCherry: 15.8 ± 1.3;<br>VGF-mCherry: 18.2 ± 0.5<br><br>Frequency<br>mCherry: 15.7 ± 3.1;<br>VGF-mCherry: 12.3 ± 2.2	[cells, slices, brains]<br>mCherry,<br>n = 9, 9, 4; VGF-mCherry,<br>n = 7, 7, 4	Two-tailed Student's t-test	Amplitude:<br>0.26<br><br>Frequency:<br>0.42
Extended Data Figure 9l	E/I ratio	mCherry: 0.35 ± 0.06;<br>VGF-mCherry: 0.43 ± 0.07	[cells, slices, brains]<br>mCherry,<br>n = 9, 9, 4; VGF-mCherry,<br>n = 7, 7, 4	Two-tailed Student's t-test	0.39
Extended Data Figure 9n	Paired-pulse ratio	mCherry: 0.46 ± 0.13;<br>VGF-mCherry: 0.33 ± 0.10	[cells, slices, brains]<br>mCherry,<br>n = 9, 9, 4; VGF-mCherry,<br>n = 10, 10, 4	Two-tailed Student's t-test	0.46
**Extended Data Figure 10**	**Measurement (unit)**	**Values**	**N [unit]**	**Statistical test**	**P-value**

Extended Data Figure 10b	Time freezing (%)	Unshocked: 9.8 ± 1.8 Shocked: 62.8 ± 5.6	[mice] Unshocked, n = 4; Shocked: n = 5	Two-tailed Student's t-test	$8x10^{-5}$
Extended Data Figure 10d	Fos-positive PV+ interneurons	41.0	[cells, brains] n = 117, 5	n.a.	n.a.
