# Supplementary material for: Regulation of PV interneuron plasticity by neuropeptide-encoding genes
Source: Nature. 2025 Apr 30;643(8070):173–81. doi: 10.1038/s41586-025-08933-z (PMC12222018; doi:10.1038/s41586-025-08933-z)
Supplement: Supplementary file 2 — Reporting Summary [file 41586_2025_8933_MOESM2_ESM.pdf]

nature portfolio

Corresponding author(s): Oscar Marín

Last updated by author(s): Jan 28, 2025

Reporting Summary

Nature Portfolio wishes to improve the reproducibility of the work that we publish. This form provides structure for consistency and transparency in reporting. For further information on Nature Portfolio policies, see our Editorial Policies and the Editorial Policy Checklist.

Statistics

For all statistical analyses, confirm that the following items are present in the figure legend, table legend, main text, or Methods section.

n/a	Confirmed	
☐	☒	The exact sample size (*n*) for each experimental group/condition, given as a discrete number and unit of measurement
☐	☒	A statement on whether measurements were taken from distinct samples or whether the same sample was measured repeatedly
☐	☒	The statistical test(s) used AND whether they are one- or two-sided<br>*Only common tests should be described solely by name; describe more complex techniques in the Methods section.*
☐	☒	A description of all covariates tested
☐	☒	A description of any assumptions or corrections, such as tests of normality and adjustment for multiple comparisons
☐	☒	A full description of the statistical parameters including central tendency (e.g. means) or other basic estimates (e.g. regression coefficient) AND variation (e.g. standard deviation) or associated estimates of uncertainty (e.g. confidence intervals)
☐	☒	For null hypothesis testing, the test statistic (e.g. *F*, *t*, *r*) with confidence intervals, effect sizes, degrees of freedom and *P* value noted<br>*Give P values as exact values whenever suitable.*
☒	☐	For Bayesian analysis, information on the choice of priors and Markov chain Monte Carlo settings
☒	☐	For hierarchical and complex designs, identification of the appropriate level for tests and full reporting of outcomes
☒	☐	Estimates of effect sizes (e.g. Cohen's *d*, Pearson's *r*), indicating how they were calculated

Our web collection on statistics for biologists contains articles on many of the points above.

Software and code

Policy information about availability of computer code

Data collection	Image acquisition was done using LAS AF software (v3.5.7.23225, Leica). Electrophysiological data was aquired using Clampex 10.7 (Molecular Devices). Mouse movement was recorded using Video Freeze (v3.1.0.0, Med Associated).
Data analysis	Image analysis was done using FIJI (1.52p) or Matlab (R2021b). Electrophysiological data was analysed using MiniAnalyis (v6.0.7, Synaptosoft) for mEPSCs, mIPSCs and sIPSCs, or Clampfit 10.2 (Molecular Devices) for all other data. RNA-sequencing reads were processed using Nextflow (v21.03.0.edge), FastQC (v0.11.9), Trim Galore (v0.6.10), STAR (2.7.10), Salmon (v0.11.9) and differential expression was analysed in R (4.2.1) using the tximport package (1.25.1) and edgeR (3.38). Mouse behavioural data was analysed using Video Freeze (v3.1.0.0). Statistical analysis was done in SPSS (v29.0.2.0.). Synapse analysis code has been previously described (DOI: 10.1126/science.aau8977). The RNA sequencing analysis pipeline is available on:

For manuscripts utilizing custom algorithms or software that are central to the research but not yet described in published literature, software must be made available to editors and reviewers. We strongly encourage code deposition in a community repository (e.g. GitHub). See the Nature Portfolio guidelines for submitting code & software for further information.

Data

Policy information about availability of data

All manuscripts must include a data availability statement. This statement should provide the following information, where applicable:

- Accession codes, unique identifiers, or web links for publicly available datasets
- A description of any restrictions on data availability
- For clinical datasets or third party data, please ensure that the statement adheres to our policy

RNA-sequencing data will be deposited into the public repository Gene Expression Omnibus (GSE223038) before publication. All other data will be shared upon reasonable request.

Human research participants

Policy information about studies involving human research participants and Sex and Gender in Research.

Reporting on sex and gender	Not applicable
Population characteristics	Not applicable
Recruitment	Not applicable
Ethics oversight	Not applicable

Note that full information on the approval of the study protocol must also be provided in the manuscript.

Field-specific reporting

Please select the one below that is the best fit for your research. If you are not sure, read the appropriate sections before making your selection.

☒ Life sciences ☐ Behavioural & social sciences ☐ Ecological, evolutionary & environmental sciences

For a reference copy of the document with all sections, see nature.com/documents/nr-reporting-summary-flat.pdf

Life sciences study design

All studies must disclose on these points even when the disclosure is negative.

Sample size	No statistical methods were used to predetermine sample sizes. Sample sizes were chosen to correspond to those reported in previous publications and generally employed in the field, e.g:<br><br>DOI: 10.1126/science.abm7466,<br>DOI: 10.1126/science.aau8977<br>DOI: 10.1038/srep34240<br><br>Each experimental condition was carried out with a minimum of three biological replicates.
Data exclusions	Electrophysiological recording were excluded if recordings did not meet pre-established criteria: Ra > 25, Ihold > -200pA or, for ChR2 evoked currents, Rs changed more than 20%. For synaptic analysis, individual cells were excluded when their soma could not be well defined and reconstructed. For RNA-sequencing analysis, genes with less than 10 reads in at least 4 samples were excluded. Outliers were defined as datapoints > 2 times SEM away from the mean and excluded.
Replication	Biological replicates were analysed to assess the biological variability and reproducibility of data. All experiments contain at least 3 biological replicates. All replicates were included when they passed quality control (see 'data exclusions').
Randomization	CNO/vehicle treatments were randomly assigned within each litter where applicable. CNO/vehicle treatment was alternated following an ABAB pattern, and mice were used for electrophysiological recordings or transcardial perfusions in pairs or multiples of pair when possible. For electrophysiological recordings, when more than 1 mice was recorded on a given day, treatment conditions were alternated. Treatment condition for the first mouse of each day was also alternated when possible.
Blinding	Electrophysiological data and tissue for RNA sequencing was not obtained blinded because of the need to inject CNO or vehicle to specific animals prior to the experiment. Image acquisition and image analysis was performed blind to the treatment condition (CNO/vehicle) of the sample.

Reporting for specific materials, systems and methods

We require information from authors about some types of materials, experimental systems and methods used in many studies. Here, indicate whether each material, system or method listed is relevant to your study. If you are not sure if a list item applies to your research, read the appropriate section before selecting a response.

Materials & experimental systems

n/a	Involved in the study
☐	☒ Antibodies
☐	☒ Eukaryotic cell lines
☒	☐ Palaeontology and archaeology
☐	☒ Animals and other organisms
☒	☐ Clinical data
☒	☐ Dual use research of concern

Methods

n/a	Involved in the study
☒	☐ ChIP-seq
☒	☐ Flow cytometry
☒	☐ MRI-based neuroimaging

Antibodies

Antibodies used

The following primary antibodies and concentrations were used: Goat anti-mCherry (1:500, Antibodies-Online, ABIN1440057), dsRed anti-rabbit (1:500, Clontech, 632496), c-Fos anti-rabbit (1:200, Merck, ABE457), Gephyrin anti-Mouse-IgG1 (1:500, Synaptic Systems, 147011), VGaT anti-Guinea pig (1:500, Synaptic Systems, 131004), Parvalbumin anti-Chicken (1:250, Synaptic Systems, 195006), Parvalbumin anti-Mouse (1:3000, Swant, 235), Synaptotagmin-2 anti-Mouse-IgG2 (1:250, ZFIN, ZDB-ATB-081002-25). The following secondary antibodies and concentrations were used: Donkey anti-Chicken 405 (1:200, Jackson, 703-475-155), Goat anti-Mouse-IgG1 488 (1:500, Molecular Probes, A21121), Donkey anti-Rabbit 488 (1:400, Thermo Fisher Scientific, A21206), Donkey anti-Rabbit 555 (1:400, Molecular Probes, A31572), Donkey anti-Goat 555 (1:400, Invitrogen, A21432), Goat anti-Mouse-IgG2 647 (1:500, Molecular Probes, A21241), Donkey anti-Guinea Pig 647 (1:250, Jackson, 706-605-148), Donkey anti-Mouse IgG1 647 (1:400, Thermo Fisher Scientific, A31571).

Validation

Goat anti-mCherry, dsRed anti-rabbit, Gephyrin anti-Mouse-IgG1,Parvalbumin anti-Chicken and Synaptotagmin-2 anti-Mouse-IgG2 have been previously used in DOI: 10.1126/science.abm7466

c-Fos anti-rabbit has previously been used in DOI: 10.1038/s41593-019-0552-7

VGaT anti-Guinea pig has previously been used in DOI: 10.1038/ncomms6066

Parvalbumin anti-Mouse has previously been used in DOI: 10.1038/s41593-018-0162-9

Eukaryotic cell lines

Policy information about cell lines and Sex and Gender in Research

Cell line source(s)	HEK293FT cells were purchased from Invitrogen
Authentication	No additional authentication was done
Mycoplasma contamination	HEK293FT cells were routinely tested for mycoplasma. All test were negative.
Commonly misidentified lines (See ICLAC register)	Not applicable

Animals and other research organisms

Policy information about studies involving animals; ARRIVE guidelines recommended for reporting animal research, and Sex and Gender in Research

Laboratory animals	All animals used in this study were Mus Musculus (House Mouse), aged P42 to P70. We used the following strains: PV-cre (B6.129P2-Pvalbtm1(cre)Arbr/J, JAX017320), CD1 (Crl:CD1[ICR], Charles River), PV-flp (B6.Cg-Pvalbtm4.1(flpo)Hze/J, JAX022730), ChR2-flx (Ai32, B6;129S-Gt(ROSA)26Sortm32(CAG-COP4*H134R/EYFP)Hze/J, JAX012569), SST-cre (Ssttm2.1(cre)Zjh/J, JAX013044), VIP-cre (Viptm1(cre)Zjh/J, JAX010908) and Nex-cre (Neurod6tm1(cre)Kan).
Wild animals	This study did not involve wild animals
Reporting on sex	Similar numbers of male and female mice were used indiscriminately in all experiments.
Field-collected samples	This study did not involve samples collected from the field.

Ethics oversight	Animal work was carried out under license from the UK Home Office following the Animals (Scientific Procedures) Act 1986.

Note that full information on the approval of the study protocol must also be provided in the manuscript.
